# Two Polyketides Intertwined in Complex Regulation: Posttranscriptional CsrA-Mediated Control of Colibactin and Yersiniabactin Synthesis in Escherichia coli

**DOI:** 10.1128/mbio.03814-21

**Published:** 2022-02-01

**Authors:** Nadine Rehm, Alexander Wallenstein, Marla Keizers, Stefan Homburg, Giuseppe Magistro, Camille V. Chagneau, Hanna Klimek, Olga Revelles, Emmanuelle Helloin, Johannes Putze, Jean-Philippe Nougayrède, Sören Schubert, Eric Oswald, Ulrich Dobrindt

**Affiliations:** a Institut für Hygiene, Universität Münster, Münster, Germany; b Interdisziplinäres Zentrum für Klinische Forschung (IZKF) Münster, Universität Münster, Münster, Germany; c Institut für Molekulare Infektionsbiologie, Universität Würzburg, Würzburg, Germany; d Urologische Klinik und Poliklinik, Ludwig-Maximilians-Universität München, Munich, Germany; e IRSD, Université de Toulouse, INSERM, INRA, ENVT, UPS, Toulouse, France; f LISBP, Université de Toulouse, CNRS, INRA, INSA, UPS, Toulouse, France; g ISP, INRA, Université de Tours, UMR 1282, Nouzilly, France; h Max von Pettenkofer-Institut für Hygiene und Medizinische Mikrobiologie, Medizinische Fakultät, Ludwig-Maximilians-Universität München, Munich, Germany; University of Texas Southwestern Medical Center Dallas

**Keywords:** secondary metabolite, cytopathic effect, BarA-UvrY, two-component regulatory systems, pathogenicity islands, high pathogenicity island

## Abstract

Bacteria have to process several levels of gene regulation and coordination of interconnected regulatory networks to ensure the most adequate cellular response to specific growth conditions. Especially, expression of complex and costly fitness and pathogenicity-associated traits is coordinated and tightly regulated at multiple levels. We studied the interconnected regulation of the expression of the colibactin and yersiniabactin polyketide biosynthesis machineries, which are encoded by two pathogenicity islands found in many phylogroup B2 Escherichia coli isolates. Comparative phenotypic and genotypic analyses identified the BarA-UvrY two-component system as an important regulatory element involved in colibactin and yersiniabactin expression. The carbon storage regulator (Csr) system controls the expression of a wide range of central metabolic and virulence-associated traits. The availability of CsrA, the key translational regulator of the Csr system, depends on BarA-UvrY activity. We employed reporter gene fusions to demonstrate UvrY- and CsrA-dependent expression of the colibactin and yersiniabactin determinants and confirmed a direct interaction of CsrA with the 5′ untranslated leader transcripts of representative genes of the colibactin and yersiniabactin operons by RNA electrophoretic mobility shift assays. This posttranscriptional regulation adds an additional level of complexity to control mechanisms of polyketide expression, which is also orchestrated at the level of ferric uptake regulator (Fur)-dependent regulation of transcription and phosphopantetheinyl transferase-dependent activation of polyketide biosynthesis. Our results emphasize the interconnection of iron- and primary metabolism-responsive regulation of colibactin and yersiniabactin expression by the fine-tuned action of different regulatory mechanisms in response to variable environmental signals as a prerequisite for bacterial adaptability, fitness, and pathogenicity in different habitats.

## INTRODUCTION

A variety of Escherichia coli strains belonging to the phylogenetic group B2, which comprises extraintestinal pathogenic E. coli (ExPEC) as well as commensal E. coli strains, have been identified to carry two potentially linked genomic islands, namely, the so-called “high pathogenicity island” (HPI) and the polyketide synthase (*pks*) island. Both islands are chromosomally located in close proximity, and the *pks* island always coexists with the HPI (1–3). Each island carries all necessary genes for a combined polyketide synthase (PKS) and nonribosomal peptide synthase (NRPS) biosynthesis machinery, producing either colibactin or yersiniabactin.

Yersiniabactin, a metallophore involved in metal homeostasis, was first discovered in Yersinia enterocolitica but was later shown to exist in members of *Enterobacterales*, such as E. coli (1, 4–7). Yersiniabactin can complex Fe(III) ions and Cu(II) ions and afterward internalize these complexes in a FyuA-dependent manner (8–11). FyuA is an HPI-encoded outer membrane protein, playing an important role in the virulence of ExPEC (12–19). Investigation of the role of the HPI in E. coli gene regulation also indicated a connection between motility and yersiniabactin expression, suggesting the interplay between the flagellar regulatory system and the promoter region of the major HPI regulator YbtA (15, 20, 21).

The *pks* island encodes the synthesis machinery required for colibactin production. Colibactin is a hybrid polyketide/nonribosomal peptide causing DNA damage in mammalian cells upon infection (22). Different colibactin structures have been suggested recently (23–26). Colibactin mediates cross-linking of DNA, *in vitro* as well as *in vivo*, resulting in DNA double-strand breaks, thus provoking cell cycle arrest and inducing megalocytosis/senescence (22, 25–27). This genotoxin promotes bacterial virulence (28, 29), but the ability to produce colibactin has also been correlated with probiotic and analgesic effects (30, 31), potential antimicrobial activity (32), and cancer propagation (33–37). We are also beginning to better understand the extent to which growth conditions and bacterial factors affect the regulation of colibactin expression in E. coli (38–41). To further investigate the regulation of expression, we screened *pks* island-positive E. coli for their ability to produce colibactin.

Generally, each PKS and/or NRPS machinery-encoding determinant includes its own phosphopantetheinyl transferase (PPTase) gene. However, in E. coli, the HPI lacks a gene coding for a PPTase, and it has been demonstrated that the *pks* island-encoded PPTase ClbA can also activate PKSs and NRPSs involved in yersiniabactin synthesis (42, 43). Moreover, gene expression of both the HPI and the *pks* island is regulated by the global iron-responsive regulatory protein ferric uptake regulator (Fur) (44, 45). It was also demonstrated that the E. coli heat shock protein HtpG is required for colibactin and yersiniabactin production (46). The co-occurrence and physical linkage of the *pks* island and the HPI, together with their common Fur-dependent regulation, the role of the HtpG chaperone for the production of colibactin and yersiniabactin, and the importance of the *pks* island-encoded PPTase ClbA for yersiniabactin expression demonstrate the close relationship between these two islands coding for secondary metabolites contributing to the fitness of extraintestinal pathogenic and commensal E. coli. Against this background, the findings that yersiniabactin expression is affected by motility regulation (15) and that uropathogenic E. coli (UPEC) model strain 536 and its spontaneous nonhemolytic pathogenicity island I and II deletion mutant 536-21 (47) appeared to be phenotypically colibactin negative despite carrying an intact *pks* island led us to further investigate the regulation of colibactin and yersiniabactin expression. We applied reporter gene constructs and electrophoretic mobility shift assays, as well as different phenotypic analyses in different strain backgrounds, to demonstrate the complex dependencies inside this regulatory network.

## RESULTS

### The response regulator UvrY is indispensable for the induction of a colibactin-mediated cytopathic effect.

**FIG 1:**
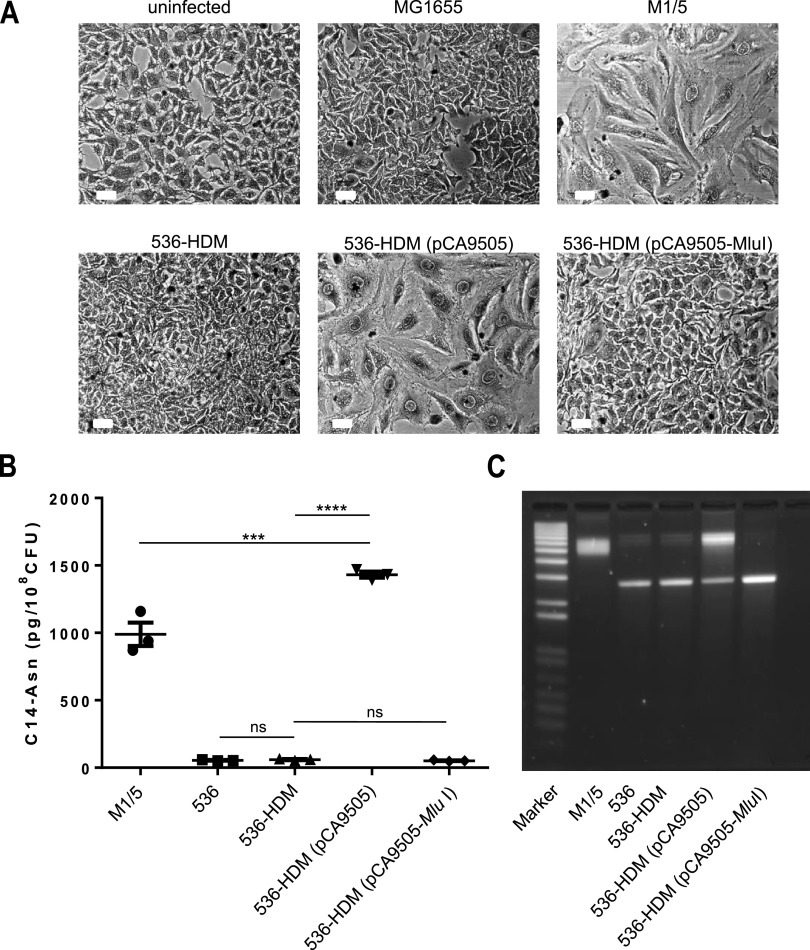
The *pks* island-positive nonhemolytic mutant E. coli 536-HDM requires exogenous UvrY to induce a cytopathic effect in HeLa cells. (A) HeLa cells were either not infected or infected with the indicated E. coli strains to a multiplicity of infection (MOI) of 200. After 4 hours of infection, HeLa cells were washed to remove bacteria and further cultivated. At 72 h postinfection, cells were washed and Giemsa stained. Scale bars, 50 μm. The *uvrY* plasmid pCA9505 (50) was used to complement the *uvrY* deletion in E. coli strain 536-HDM. As a control, E. coli strain 536-HDM was transformed with the *uvrY*-negative variant of plasmid pCA9505, namely, pCA9505-*MluI* (90). (B) *N*-Myristoyl-d-asparagine (C14-asparagine) quantification (mean values ± SEM) in bacterial cultures of E. coli strains M1/5, 536, 536-HDM, 536-HDM (pCA9505), and 536-HDM (pCA9505-*MluI*) by liquid chromatography-tandem mass spectrometry (LC-MS/MS). The data presented in the graph were obtained from three biological replicates. ****, *P* < 0.0001; ***, *P* < 0.001; **, *P* < 0.01; 1-way ANOVA with Bonferroni correction. (C) DNA cross-link formation of plasmid DNA exposed to strains M1/5, 536, 536-HDM, 536-HDM (pCA9505), and 536-HDM (pCA9505-*MluI*) was visualized after migration under alkaline denaturing conditions. M, DNA size marker (1 kb plus DNA ladder, Invitrogen). This image is representative of three independent experiments.

Megalocytosis assays with the nonhemolytic mutant of phylogroup B2 UPEC strain E. coli 536, designated 536-HDM (48), demonstrated that despite the presence of an intact *pks* island, a cytopathic effect (CPE) could not be observed upon infection (Fig. 1A). As colibactin is first synthetized as a prodrug (so-called precolibactin), carrying an *N*-myristoyl-d-asparagine (C14-asparagine [C14-asn]) side chain which is cleaved in the periplasm to release the active genotoxin, C14-asn can be used as a readout for colibactin expression. Interestingly, E. coli strains 536 and 536-HDM displayed a low level of C14-asn production as well as a weak DNA cross-link activity (Fig. 1B and C). This finding suggests that the *pks* island is functional but only weakly expressed in E. coli strain 536. Accordingly, E. coli 536-HDM infected HeLa cells showed a similar cell size as noninfected HeLa cells or HeLa cells incubated with E. coli K-12 strain MG1655 (Fig. 1A), whereas HeLa cell infection with *pks*-positive phylogroup B2 E. coli isolate M1/5 led to megalocytosis (Fig. 1A) and high levels of *N*-myristoyl-d-asparagine production, as well as a significantly stronger DNA cross-link activity (Fig. 1B and C). A genome sequence analysis of E. coli strain 536 revealed that the *pks* island was fully conserved in this strain, but we detected, in comparison to the published colibactin-producing newborn meningitis isolate IHE3034 as well as to K-12 laboratory strain MG1655, a 6.1-kb chromosomal deletion between *uvrC* and *fliY* (see Fig. S1 in the supplemental material), which also includes the *uvrY* gene encoding the response regulator UvrY of the BarA-UvrY two-component regulatory system (TCS) (49). We, therefore, assumed that the presence of UvrY is crucial for the colibactin-mediated phenotypic effect. To test this hypothesis, *uvrY* plasmid pCA9505 (50) was used to complement the *uvrY* deletion in E. coli strain 536-HDM. Infection with this complemented strain led to the induction of megalocytosis in HeLa cells, high C14-asn production, and DNA cross-linking activity (Fig. 1A to C) confirming our initial assumption that strain 536 codes for a functional colibactin biosynthesis machinery, which requires UvrY for its proper expression. Transformation of E. coli 536-HDM with a vector control (pCA9505-*MluI*) did not cause megalocytosis upon HeLa infection. Also, the production of C14-asn and DNA cross-linking activity were not increased compared with those of wild-type strain 536 and its nonhemolytic mutant 536-HDM (Fig. 1A to C).

10.1128/mbio.03814-21.1 FIG S1 Comparison of the genomic region comprising *uvrY* in different E. coli strains. Analysis of the chromosomal region next to *uvrY* in the complete genome sequences of *pks* island-positive, but CPE-negative, uropathogenic E. coli strain 536 with those of colibactin-producing newborn meningitis isolate IHE3034 as well as to K-12 laboratory strain MG1655 that indicated that E. coli 536 carries a 6.1-kb chromosomal deletion spanning the region between *uvrC* and *fliY* and thus lacks the *uvrY* gene. Download FIG S1, PDF file, 0.2 MB. Copyright © 2022 Rehm et al. 2022 Rehm et al. https://creativecommons.org/licenses/by/4.0/ This content is distributed under the terms of the Creative Commons Attribution 4.0 International license.

**FIG 2:**
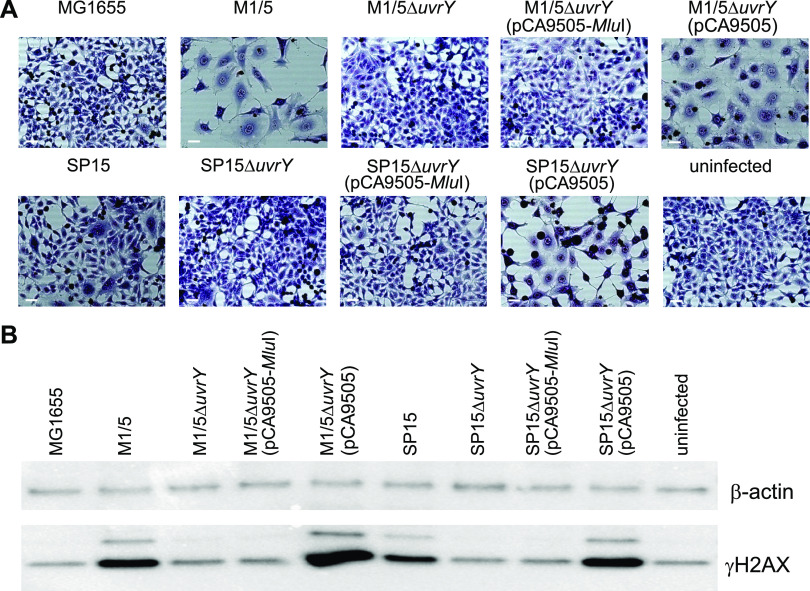
UvrY is indispensable for the efficient induction of double-strand breaks and megalocytosis by *pks*-positive E. coli strains. (A) HeLa cells were either infected with the indicated E. coli strains (MOI of 200) or not infected. After 4 hours of infection, HeLa cells were washed to remove bacteria and further cultivated. At 72 h postinfection, cells were washed and Giemsa stained. Scale bars, 50 μm. (B) At 8 h postinfection, cells were washed with phosphate-buffered saline (PBS) and lysed. An amount of 5 μg total protein per lane of indicated samples were analyzed by SDS-PAGE and afterward transferred onto a polyvinylidene difluoride (PVDF) membrane. γH2AX was detected using the anti-gammaH2A.X (phospho S139) antibody (Abcam). β-Actin served as a loading control.

Next, we wanted to investigate if the UvrY dependency of the colibactin-mediated phenotype was restricted to E. coli strain 536. As shown in Fig. 2A, HeLa cell infection with *pks*-positive E. coli wild-type strains M1/5 and SP15 resulted in strong megalocytosis. Moreover, infection with these strains led to the formation of high γH2AX levels as an indicator of DNA double-strand breaks in mammalian cells (Fig. 2B). As described above, colibactin induces DNA double-strand breaks, which are accompanied by the phosphorylation of the small histone H2AX at serine residue 139. However, infection with *uvrY* deletion mutants E. coli M1/5Δ*uvrY* and SP15Δ*uvrY* abrogated the CPE since no megalocytosis and only low levels of γH2AX could be detected that were comparable to HeLa cell mock infection or infection with E. coli K-12 strain MG1655. Transformation of E. coli strains M1/5Δ*uvrY* and SP15Δ*uvrY* with plasmid-encoded *uvrY* using pCA9505 led to the reconstitution of the CPE as indicated by the appearance of strong γH2AX signals and detection of enlarged cells. In contrast, transformation with the *uvrY*-negative variant pCA9505-*MluI* did not lead to the reconstitution of the CPE. Taken together, these results clearly show that, although the colibactin determinant is generally expressed at a very low level, the presence of UvrY is essential for efficient colibactin expression.

### The carbon storage regulator (Csr) system is required for the colibactin-mediated cytopathic effect.

**FIG 3:**
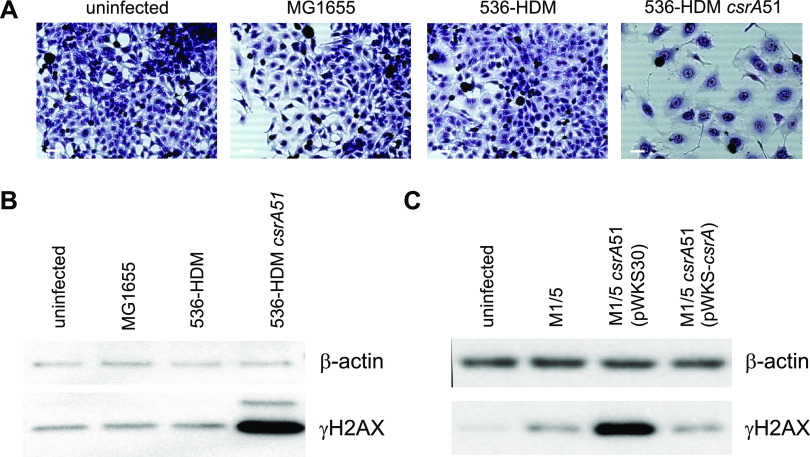
CsrA blocks the colibactin-mediated cytopathic effect of *pks*-positive E. coli strains. (A) HeLa cells were either not infected or infected with the indicated E. coli strains (MOI of 200). After 4 hours of infection, HeLa cells were washed to remove bacteria and further cultivated. At 72 h postinfection, cells were washed and Giemsa stained. Scale bars, 50 μm. Eight hours postinfection, cells were washed with PBS and lysed. A total protein amount of 5 μg (B) or 4 μg (C) per lane of indicated samples was analyzed by SDS-PAGE and afterward transferred onto PVDF membranes. γH2AX was detected using either an anti-gammaH2A.X (phospho S139) antibody (Abcam) (B) or anti-phospho-Histone H2A.X (Ser139), clone EP854(2)Y (Merck-Millipore) (C). β-Actin served as a loading control.

The BarA-UvrY TCS regulates the synthesis of the small noncoding RNAs *csrB* and *csrC*, which antagonize the function of the global RNA-binding protein CsrA (51). Since UvrY activates *csrB* and *csrC* transcription, we hypothesized that deletion of *csrB* and *csrC* would also lead to abrogation of the CPE. Several deletion mutants as well as the corresponding complemented mutants of newborn meningitis E. coli isolate SP15 were generated and tested for their ability to cause megalocytosis in HeLa cells. This E. coli strain has been described previously as evoking colibactin-dependent CPE in mammalian cells (22). Single deletion of either *csrB* or *csrC* in E. coli strain SP15 did not lead to an abrogation of the cytotoxic effect, whereas the double deletion mutant SP15Δ*csrB*Δ*csrC* was not able to cause megalocytosis in HeLa cells, indicating that at least one of the two noncoding RNAs is required for proper expression of the colibactin-mediated megalocytosis phenotype. Transformation of this strain with plasmid pRS-*csrB* reconstituted its ability to cause a CPE (see Fig. S2 in the supplemental material; Table 1). The observation that the *csrB* and *csrC* small noncoding regulatory RNAs can complement each other’s function has already been described (52, 53). Accordingly, the regulatory RNAs *csrB* and *csrC* regulated by the BarA-UvrY TCS are required in *pks*-positive strains to evoke a CPE on mammalian cells. The *csrB* and *csrC* RNAs sequester and thus antagonize the function of the key translational regulator CsrA (54). To test whether colibactin synthesis and/or delivery was repressed by CsrA, we partially deleted *csrA* in E. coli strain 536-HDM resulting in the production of a truncated CsrA protein, CsrA51, with reduced functionality (54). While HeLa cells infected with E. coli strains MG1655 or 536-HDM behaved like noninfected cells and neither led to megalocytosis (Fig. 3A) nor γH2AX formation (Fig. 3B), incubation of HeLa cells with the partial *csrA* deletion mutant E. coli 536-21*csrA*51 led to the detection of γH2AX as well as megalocytotic cells (Fig. 3A). The same effect was detected upon *csrA* truncation and complementation of this mutant in E. coli strain M1/5 (Fig. 3C). Thus, CsrA is a repressor of colibactin synthesis and/or the colibactin-mediated CPE.

**TABLE 1:** Effect of *csrB* and *crsC* deletions in E. coli strains on the colibactin-mediated CPE in HeLa cells

Strain	Megalocytosis phenotype
SP15	+
SP15 Δ*csrB*	+
SP15 Δ*csrC*	+
SP15 Δ*csrB* Δ*csrC*	−
SP15 Δ*csrB* Δ*csrC* pRS-*csrB*	+

10.1128/mbio.03814-21.2 FIG S2 Impact of the small regulatory RNAs *csrB* and *csrC* on the cytopathic effect of E. coli strain SP15 in HeLa cells. HeLa cells were either not infected or infected with the indicated E. coli strains to a multiplicity of infection (MOI) of 250. After 4 hours of infection, HeLa cells were washed to remove bacteria and further cultivated. At 72 h postinfection, cells were washed and Giemsa stained. Scale bars, 100 μm. Plasmid pRS-*csrB* was used to complement the *csrB* and *csrC* deletion in E. coli strain SP15Δ*csrB* Δ*csrC*. Download FIG S2, PDF file, 0.4 MB. Copyright © 2022 Rehm et al. 2022 Rehm et al. https://creativecommons.org/licenses/by/4.0/ This content is distributed under the terms of the Creative Commons Attribution 4.0 International license.

### CsrA directly represses the synthesis of enzyme(s) encoded by the *pks* island.

Since we demonstrated in different E. coli strain backgrounds that CsrA is involved in the colibactin synthesis and/or the colibactin-mediated host cell damage, we investigated whether CsrA directly represses the expression of colibactin genes. A SELEX-derived consensus motif from 55 ligands for E. coli CsrA binding has been published (55, 56). The motif was determined as RUACARGGAUGU, with the underlined sequence being 100% conserved and the R representing a purine base. When we performed a bioinformatics search for respective motifs covering the nucleotide sequence ACARGGA within the 19 genes of the *pks* island of E. coli M1/5, we detected 7 motifs of putative CsrA binding sequences (Table 2).

**TABLE 2:** Sequences in the *pks* island with a putative CsrA binding motif

Location	Sequence^*a*^
Within *clbG*	GAACAGGGATTT
Within *clbI*	TCACAGGGACGT
Within *clbJ*	TCACAGGGATGT
Directly upstream of *clbL*	CAACAGGGAGAA
Within *clbN*	TGACAAGGAGAA
Directly upstream of *clbQ*	CAACAAGGAGTG
Directly upstream of *clbS*	ATACAAGGAGCA

a Nucleotides, which are 100% conserved in the consensus motif described by Dubey and colleagues, (55) are underlined.

**FIG 4:**
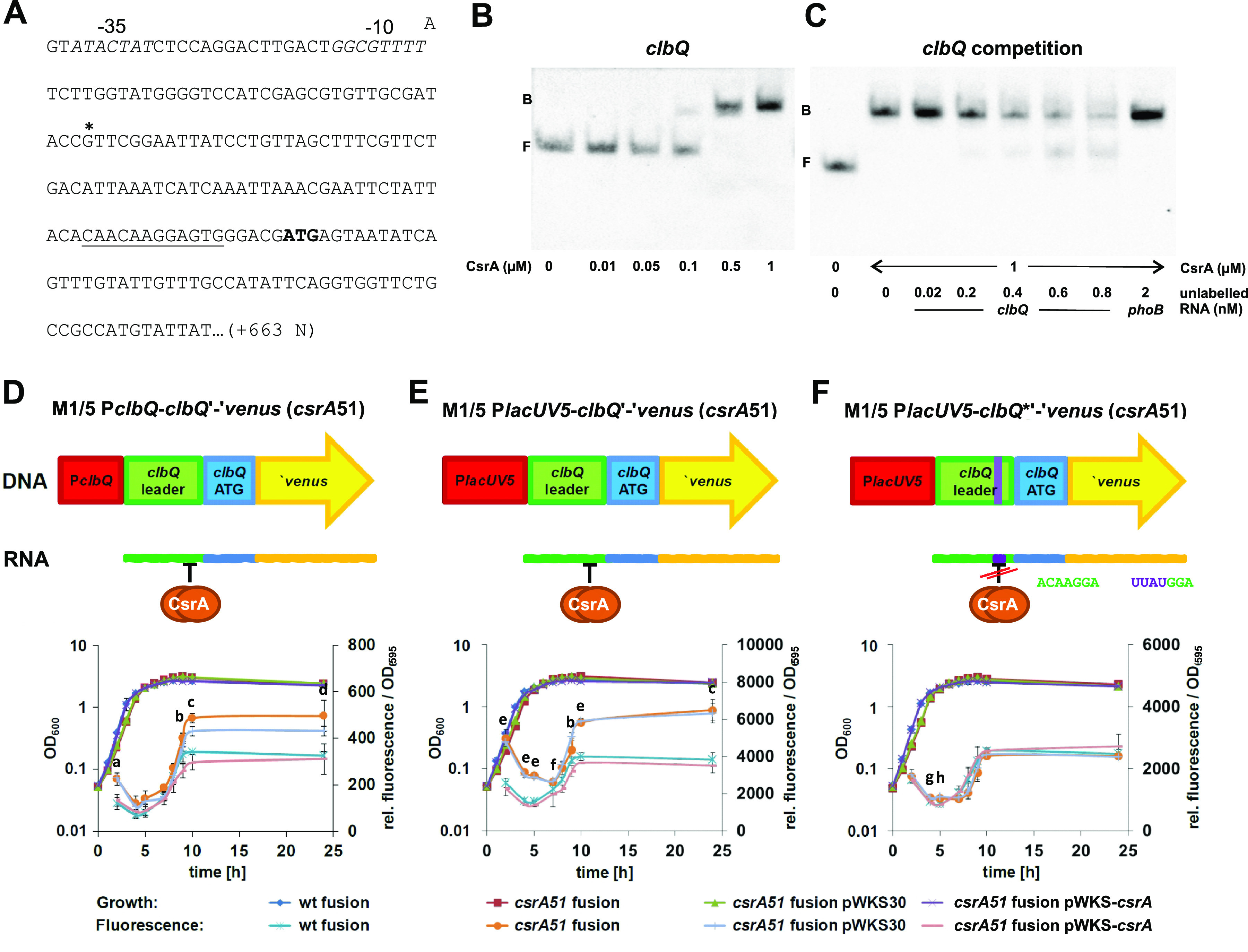
Expression of *clbQ* is directly repressed by CsrA in E. coli strain M1/5. (A) The partial sequence of the *clbQ* locus is shown, including the −35 and −10 regions (in italics) and the transcriptional start (*). The putative CsrA-binding motif is underlined, and the start codon for translation is indicated in bold. The sequence from the transcription start downstream corresponds to the sequence used for *clbQ* RNA probe generation used in RNA electrophoretic mobility shift assays (EMSAs) shown in Fig. 4B and C. (B) An RNA EMSA with a biotin-labeled *clbQ* RNA probe (20 pM) and increasing amounts of purified CsrA protein (10 nM, 50 nM, 100 nM, 500 nM, and 1 μM; F, free probe; B, bound probe) was performed. The sequence from the transcription start corresponds to the sequence used for *clbQ* RNA probe generation. (C) Biotin-labeled *clbQ* RNA probe (20 pM) was incubated with 1 μM CsrA and increasing amounts of unlabeled *clbQ* RNA (20 pM, 200 pM, 400 pM, 600 pM, and 800 pM; f.p., free probe) or *phoB* RNA (2 nM) in a competitive RNA EMSA. (D to F) Growth and fluorescence of a set of E. coli M1/5 versus E. coli M1/5 *csrA51* reporter gene fusion strains with *venus* as a reporter gene in a translational fusion (D), with the *lacUV5* promoter instead of the native *clbQ* promoter (E), and with the *lacUV5* promoter containing a modified sequence within the putative CsrA-binding motif (F) were monitored in M9 minimal medium without glucose but containing pyruvate and casein hydrolysate for 24 h at the indicated time points. Means and standard deviations of three independent experiments are shown for the fluorescence values. Growth curves of only one experiment are depicted since growth was essentially the same in all three experiments. Statistical analyses using the 1-way ANOVA test were performed comparing the fluorescence of E. coli M1/5 P*clbQ*-*clbQ*'-'*venus* to E. coli M1/5 *csrA*51 P*clbQ*-*clbQ*'-'*venus* (group 1) and E. coli M1/5 *csrA*51 P*clbQ*-*clbQ*'-*'venus* pWKS30 to E. coli M1/5 *csrA*51 P*clbQ*-*clbQ*'-'*venus* pWKS*csrA* (group 2) for each time point. Small letters in D, E, and F correspond to significance values of group1/group 2 as follows: a, *P* < 0.01/*P* < 0.01; b, ns/*P* < 0.01 (b); c, *P* < 0.001/*P* < 0.001; d, *P* < 0.01/*P* < 0.05; e, *P* < 0.0001/*P* < 0.0001; f, *P* < 0.01/*P* < 0.0001; g, *P* < 0.05/*P* < 0.01; h, *P* < 0.05/*P* < 0.05. If not marked, no significance was found.

The *clbQ* gene was among the genes comprising a putative CsrA binding motif in its 5′ untranslated leader sequence (Fig. 4A). ClbQ is a thioesterase and one of the last enzymes of the colibactin production machinery responsible for the hydrolytic cleavage of precolibactin from polyketides. We examined a putative interaction of *in vitro*-transcribed *clbQ* RNA with CsrA in an RNA electrophoretic shift assay (RNA EMSA). When 3′ biotin-labeled *clbQ* RNA was incubated with increasing amounts of purified CsrA protein, a clear shift of the RNA band was observed (Fig. 4B). The addition of an excess amount of unlabeled *clbQ* RNA as a competitor reverted the band shift but not 100-fold excess of *phoB* RNA, which was used as a negative control (57) (Fig. 4C). These results suggest a specific and direct interaction of CsrA with the *clbQ* untranslated leader RNA *in vitro.*

To test whether CsrA repressed *clbQ* expression, a set of reporter gene fusions were generated in E. coli strain M1/5 using the *venus* gene as a reporter. The *clbQ* locus was chromosomally manipulated either in the E. coli M1/5 wild type or in the mutant strain E. coli M1/5 *csrA*51 with a less functional CsrA protein. The growth and fluorescence of the resulting reporter strains were examined for 24 h. Fluorescence of the translational *clbQ*-*venus* fusion strain with a truncated CsrA protein, E. coli M1/5 P*clbQ*-*clbQ*'-'*venus csrA*51, was elevated about 1.6-fold compared with that of the wild-type strain M1/5 P*clbQ*-*clbQ*'-'*venus* after 10 h of growth (Fig. 4D). Bacterial growth remained almost unaffected by *csrA* manipulation throughout the experiment. However, complementation with plasmid-encoded CsrA using pWKS-*csrA*, but not with empty vector control pWKS30, resulted in fluorescence comparable to the wild type. These results confirmed that CsrA represses *venus* (*clbQ*) expression. A similar effect was observed when the *clbQ* promoter of the translational *clbQ*-*venus* fusion strains was exchanged by the constitutive *lacUV5* promoter (Fig. 4E), where *venus* fluorescence of E. coli M1/5 P*lacUV5*-*clbQ*'-'*venus csrA*51 was also increased 1.6-fold compared with that of M1/5 P*lacUV5*-*clbQ*'-'*venus* after 10 h of growth. Therefore, repression of *venus* expression by CsrA seemed to be independent of the *clbQ* promoter but was probably attributed to the presence of the predicted CsrA binding site in the *clbQ* leader sequence. To further test this hypothesis, the sequence encoding the putative CsrA binding motif of the reporter construct with the *lacUV5* promoter was genetically modified from CAACAAGGAGTG to CATTATGGAGTG. The resulting strains M1/5 P*lacUV5*-clbQ*'-'*venus* and M1/5 P*lacUV5*-clbQ*'-'*venus csrA*51 exhibited similar fluorescence throughout growth (Fig. 4F). Accordingly, alteration of the CsrA binding motif resulted in the abrogation of the inhibitory effect of CsrA on *venus* expression. We also compared the fluorescence of another set of reporter strains, namely, E. coli strains M1/5 P*clbQ*-AL-*venus* and M1/5 P*clbQ*-AL-*venus csrA*51, in which the native *clbQ* promoter was fused to a sequence encoding an artificial 5′ untranslated leader (AL) without the CsrA binding motif (see Fig. S3A in the supplemental material) after 10 h of growth. Fluorescence of both strains was comparable (Fig. S3B), confirming again that CsrA did not influence *clbQ* promoter activity and acted only on the 5′ untranslated *clbQ* leader sequence. Taken together, we demonstrate that CsrA represses *clbQ* expression directly by binding to the *clbQ* untranslated RNA leader.

10.1128/mbio.03814-21.3 FIG S3 Activity of the *clbQ* promoter fused to a sequence encoding an artificial 5′ untranslated leader and *venus* as a reporter gene. (A) Sequences encoding the *clbQ* 5′ leader and the artificial leader are shown in italics, an asterisk represents the transcription start, and the translational start codon is depicted in bold. The sequence encoding the putative CsrA-binding motif is underlined. (B) Expression from the *clbQ* promoter in E. coli strains M1/5 P*clbQ*-AL-'*venus* and M1/5 P*clbQ*-AL-'*venus* was measured by means of venus fluorescence after 10 h of growth in M9 minimal medium without glucose but containing pyruvate and casein hydrolysate. Mean values with standard deviations of three independent experiments are shown. Download FIG S3, PDF file, 0.2 MB. Copyright © 2022 Rehm et al. 2022 Rehm et al. https://creativecommons.org/licenses/by/4.0/ This content is distributed under the terms of the Creative Commons Attribution 4.0 International license.

### The Csr system influences yersiniabactin levels.

Coregulation of the expression of two probably coselected fitness determinants makes sense, and consequently, we assumed that the Csr system is involved not only in the regulation of the *pks* island-encoded polyketide colibactin but also in that of the HPI-encoded polyketide yersiniabactin.

**FIG 5:**
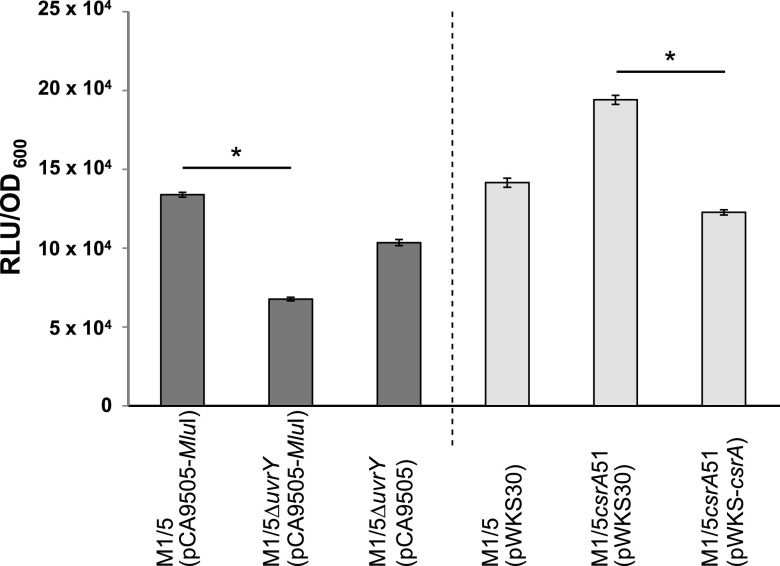
CsrA and UvrY affect yersiniabactin levels in E. coli strain M1/5. Yersiniabactin levels were quantified in the supernatants of indicated E. coli M1/5 strains grown for 24 h under iron-limiting conditions using Salmonella reporter strain WR1542. The means of three experiments are shown with standard deviations. RLU, relative light units; *, *P* < 0.05.

In the absence of UvrY, *pks*-positive E. coli isolates were not able to cause the cytopathic effect on HeLa cells, and we showed that CsrA was responsible for the repression of colibactin expression. To investigate the influence of UvrY and CsrA on yersiniabactin expression, we employed a luciferase reporter system to indirectly quantify yersiniabactin levels produced by E. coli strain M1/5 carrying or lacking either *uvrY* or a full-length *csrA* gene, respectively. Since yersiniabactin is synthesized only when iron availability is limited, quantification was performed with bacteria grown under iron-limiting conditions by adding 2,2′-dipyridyl as an iron-chelating agent. As shown in Fig. 5, yersiniabactin levels in E. coli strain M1/5 Δ*uvrY* carrying pCA9505-*MluI* were significantly reduced compared with those in its *uvrY*-positive counterpart E. coli M1/5 (pCA9505-*MluI*). Transformation of the *uvrY* deletion mutant with the *uvrY*-harboring plasmid pCA-9505 resulted in the production of yersiniabactin in amounts almost comparable to that of the wild-type strain with the empty plasmid, namely, E. coli M1/5 (pCA9505-*MluI*). Because of the negative influence of CsrA on colibactin production, we expected a similar effect of CsrA on yersiniabactin biosynthesis. Indeed, E. coli strain M1/5 *csrA*51 (pWKS30), which expresses the truncated and thus less functional CsrA protein, produced more yersiniabactin than E. coli strain M1/5 (pWKS30). When strain M1/5 *csrA*51 was complemented with the *csrA*-harboring plasmid pWKS-*csrA*, yersiniabactin synthesis was restored to the wild-type level. Thus, we show that CsrA represses phenotypic expression not only of colibactin but also of yersiniabactin.

### CsrA directly represses the synthesis of YbtA encoded by the high pathogenicity island.

**FIG 6:**
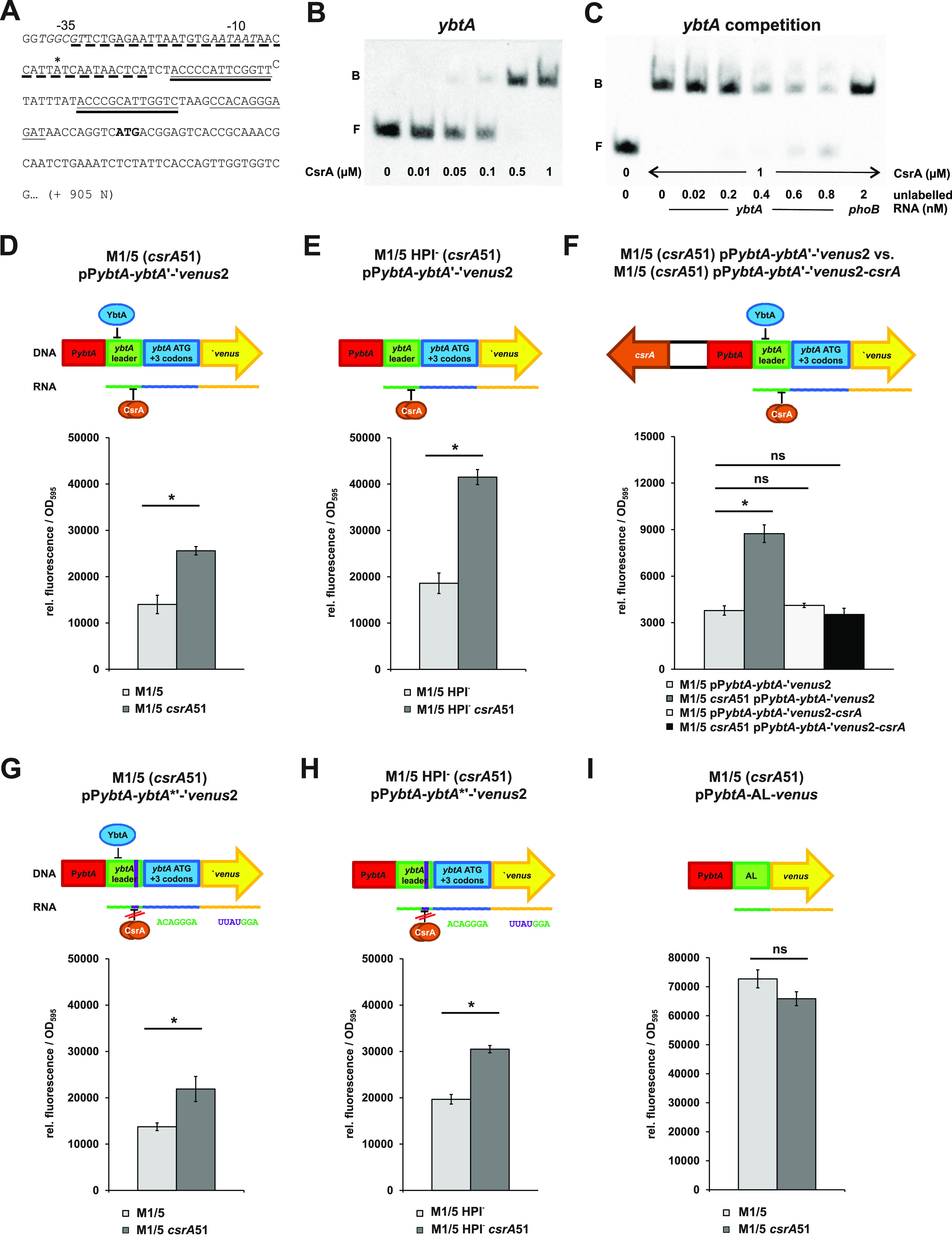
CsrA affects the expression of *ybtA* in E. coli strain M1/5. (A) Partial sequence of the *ybtA* locus with its −35 and −10 regions (in italics) and the transcriptional start site (*). Nucleotides comprising the Fur-binding motif are underlined by a dotted line, whereas YbtA-binding sites are underlined twice. The sequence encoding the putative CsrA-binding motif is highlighted by a continuous line and the translation start codon is shown in bold. The sequence depicted starting from the transcription start was used for *ybtA* RNA probe generation used in RNA EMSA experiments. (B) The biotin-labeled *ybtA* RNA probe (20 pM) was incubated without or with increasing amounts of purified CsrA protein (10 nM, 50 nM, 100 nM, 500 nM, and 1 μM; F, free probe; B, bound probe). (C) A competitive RNA EMSA was performed with 20 pM of biotin-labeled *ybtA* RNA probe, 1 μM CsrA, and increasing amounts of unlabeled *ybtA* RNA (20 pM, 200 pM, 400 pM, 600 pM, and 800 pM; f.p., free probe) or *phoB* RNA (2 nM). (D to I). Plasmid-based *ybtA-venus* reporter gene fusions were generated, and the fluorescence (relative fluorescence referred to optical density at 595 nm [OD_595_]) between *csrA*-positive E. coli M1/5 and E. coli M1/5 *csrA*51 background strains after 20 h of cultivation was compared. For this comparison, cells were grown in M9 minimal medium without glucose but containing pyruvate and casein hydrolysate. 2,2′-Dipyridyl was added to relieve Fur-mediated repression of the yersiniabactin operon. D and E show fluorescence of E. coli strains M1/5 and M1/5 *csrA*51 or E. coli M1/5 HPI^−^ and M1/5 HPI^−^ *csrA*51, respectively, when transformed with a plasmid carrying a translational *ybtA-venus* fusion with the native *ybtA* promoter and leader sequence. (F) Fluorescence of E. coli strains M1/5 and M1/5 *csrA*51 transformed with plasmids containing the translational *ybtA-venus* fusion only, pP*ybtA*-*ybtA*'-'*venus*2, or carrying a *csrA* locus in addition for complementation, pP*ybtA*-*ybtA*'-'*venus*2-*csrA*. G and H show the fluorescence of E. coli strains M1/5 and M1/5 *csrA*51 or E. coli M1/5 HPI^−^ and M1/5 HPI^−^ *csrA*51, respectively, when transformed with a plasmid carrying the translational *ybtA-venus* fusion with a sequence modification leading to a disrupted CsrA-binding motif. (I) Fluorescence of E. coli strains M1/5 and M1/5 *csrA*51 carrying a transcriptional *venus* fusion comprising the *ybtA* promoter fused to a sequence encoding an artificial 5′ leader (AL) and *venus*. *, *P* < 0.05; ns, not significant.

We assumed that CsrA might directly repress the production of proteins required for yersiniabactin synthesis. Therefore, we screened for putative CsrA binding motifs within the HPI as described for the colibactin-encoding *pks* island using the 100% conserved binding motif sequence ACARGGA (55). As indicated in Table 3, we detected seven sites comprising putative CsrA binding sequences within the HPI. In the case of *ybtA*, this motif is located in their 5′ untranslated leader sequences. The *ybtA* upstream region, including a putative CsrA binding motif and two YbtA binding sites, is shown in Fig. 6A. First, we investigated by RNA EMSA whether the 5′ *ybtA* leader sequence was bound by purified CsrA. The *ybtA* probe comprising the sequence from the *ybtA* transcription start as well as the YbtA and Fur binding sites (Fig. 6A) showed a clear retardation of migration in RNA EMSA upon incubation with increasing amounts of purified CsrA protein (Fig. 6B). The addition of excess unlabeled *ybtA* RNA to compete for CsrA binding led to a marked reduction of the shift, whereas the addition of 100-fold excess *phoB* RNA (negative control) did not reduce the RNA shift (Fig. 6C).

**TABLE 3:** Sequences in the high pathogenicity island with a putative CsrA-binding motif

Location	Sequence^*a*^
Within *ybtS*	AAACAAGGATGC
Directly upstream of *ybtA*	CCACAGGGAGAT
Within *irp2*	CCACAAGGACAA
Within *irp1*	CGACAAGGATGG
Within *ybtT*	CCACAAGGACTG
Directly upstream of *fyuA*	TTACAGGGACTC
Within *fyuA*	CCACAGGGAACG

a Nucleotides, which are 100% conserved in the consensus motif described by Dubey and colleagues (55), are underlined.

Several plasmid-based *ybtA-venus* reporter fusions were generated to compare *ybtA* promoter activity in the E. coli M1/5 wild type and its isogenic mutant M1/5 *csrA51*. When E. coli strains M1/5 and M1/5 *csrA*51 were transformed with the *ybtA* translational fusion plasmid pP*ybtA*-*ybtA*'-'*venus*2, a significant increase of *venus* expression could be observed in the mutant expressing the truncated, less functional CsrA protein (Fig. 6D), even though YbtA-mediated repression of the fusion still occurred due to the presence of a functional yersiniabactin-encoding gene cluster. CsrA-dependent repression of *ybtA* promoter activity was even more pronounced upon relief of YbtA-mediated repression when we compared fluorescence resulting from the same plasmid in E. coli strains M1/5 HPI- and M1/5 HPI- *csrA*51, which carry a partial deletion of the HPI, including *ybtA* (Fig. 6E). Introduction of a fully functional *csrA* gene into pP*ybtA*-*ybtA*'-'*venus*2, resulting in plasmid pP*ybtA*-*ybtA*'-'*venus*2-*csrA*, restored functional CsrA expression in E. coli M1/5 *csrA*51, thus reducing *ybtA* promoter activity to a similar level as that in the *csrA*-positive strain M1/5 (pP*ybtA*-*ybtA*'-'*venus*2) (Fig. 6F).

Next, we examined if CsrA repressed *ybtA* promoter activity by direct interaction with the putative CsrA binding motif present in the 5′ untranslated *ybtA* leader (see Fig. 6A). Similar to our experiments on *clbQ* expression, the putative CsrA motif CCACAGGGAGAU was therefore genetically modified to CCUUAUGGAGAU, which should interfere with proper CsrA binding. When *venus* expression was compared in the *ybtA*-positive (M1/5 versus M1/5*csrA*51) or *ybtA*-negative (M1/5 HPI- versus M1/5 HPI-*csrA*51) strain pairs using plasmid pP*ybtA*-ybtA*'-'*venus*2, fluorescence was increased in variants expressing the truncated CsrA protein (Fig. 6G or H, respectively). Disorganization of the putative CsrA binding motif in plasmid pP*ybtA*-ybtA*'-'*venus*2 diminished the fold increase in *venus* expression due to *csrA* truncation. Expression of *venus* increased upon *csrA* truncation by 1.59-fold (*ybtA*-positive strain background) and 1.55-fold (*ybtA*-negative strain background). In contrast, in the presence of an intact YbtA binding site, the reporter gene expression was 1.83-fold (*ybtA*-positive strains) and 2.23-fold (*ybtA*-negative strains) higher upon truncation of *csrA* (data used for calculation based on data shown in Fig. 6D and G and Fig. 6E and H, respectively). Reporter gene assays with the *ybtA-venus* fusion plasmid pP*ybtA*-AL-*venus* that carried the *ybtA* promoter fused to an artificial leader without CsrA binding motif (AL) (see Fig. S2) demonstrated that in the absence of a CsrA binding motif no significant difference in fluorescence was detected between the strains M1/5 and M1/5 *csrA*51 (Fig. 6I). Accordingly, the *ybtA* promoter activity was not affected by CsrA itself. Our results show that *ybtA* expression is repressed by CsrA. This effect is only partially due to the direct interaction of CsrA with the *ybtA* leader. Consequently, another so far unidentified CsrA-governed mechanism seems to be involved in the control of *ybtA* expression.

## DISCUSSION

The multilayered intertwining of two determinants encoding secondary metabolites with different functions in E. coli is remarkable. Secondary metabolite production is an energy-demanding process that also requires the provision of certain primary metabolic precursors, such as acyl-coenzyme A (CoA) moieties. Tight and coordinated regulation of secondary metabolite production is thus a way to minimize the production costs and to ensure that the bacterial producer can flexibly meet the challenges under different growth conditions and in different habitats. The observation that the *pks* island is always accompanied by the HPI in phylogroup B2 E. coli strains was made quite soon after the colibactin island was first described (1). It has also been shown that the HPI including flanking chromosomal regions can be horizontally transferred as a larger DNA entity by F-plasmid-mediated mobilization and subsequent recombination (58). Although HPI is not self-transferable, comparative genomic analyses and conjugation experiments indicated that joint horizontal transfer of the HPI can occur along with the *pks* island and another adjacent chromosomal island (59). In other colibactin-producing coliform enterobacteria, such as Klebsiella pneumoniae, Citrobacter koseri, and Klebsiella (formerly Enterobacter) aerogenes, the colibactin and yersiniabactin determinants are colocalized within an integrative and conjugative element (ICE) (1–3), confirming that physical linkage of the colibactin and yersiniabactin islands is actively supported by joint acquisition or spread via horizontal gene transfer.

The *pks* island and the HPI are not only physically linked but also interconnected at different regulatory levels of gene expression. The expression of both polyketides responds to iron availability. The ferric uptake regulator (Fur) is involved in the regulation of colibactin and yersiniabactin expression (4, 44, 45, 60). Fur represses the transcription of the small regulatory RNA *ryhB* (61). As a result, colibactin expression is also regulated at the posttranscriptional level (43, 45). The *ryhB* regulatory RNA has been shown to repress the expression of the serine acetyltransferase CysE, which facilitates the channeling of serine as a building block into enterobactin synthesis (62). Serine is also a building block of colibactin and yersiniabactin. Accordingly, the effect of *ryhB* on the expression of colibactin and yersiniabactin is not only due to an iron-dependent regulation of gene expression but also most likely on increased efficiency of nonribosomal peptide biosynthesis. In addition, the intertwining of the biosynthesis pathways of different siderophores, such as enterobactin and yersiniabactin, with colibactin has been demonstrated by showing that the PPTase ClbA, encoded by the colibactin gene cluster, can contribute not only to the synthesis of colibactin but also to the synthesis of yersiniabactin (42). This functional interchangeability of the ClbA PPTase is facilitated by the physical and regulatory linkage of both islands. Another aspect of the interplay between the biosynthesis pathways of the two polyketides is the involvement of the chaperone HtpG and the protease ClpQ. The heat shock protein HtpG appears to protect proteins involved in colibactin and yersiniabactin production from ClpQ-mediated degradation (46).

**FIG 7:**
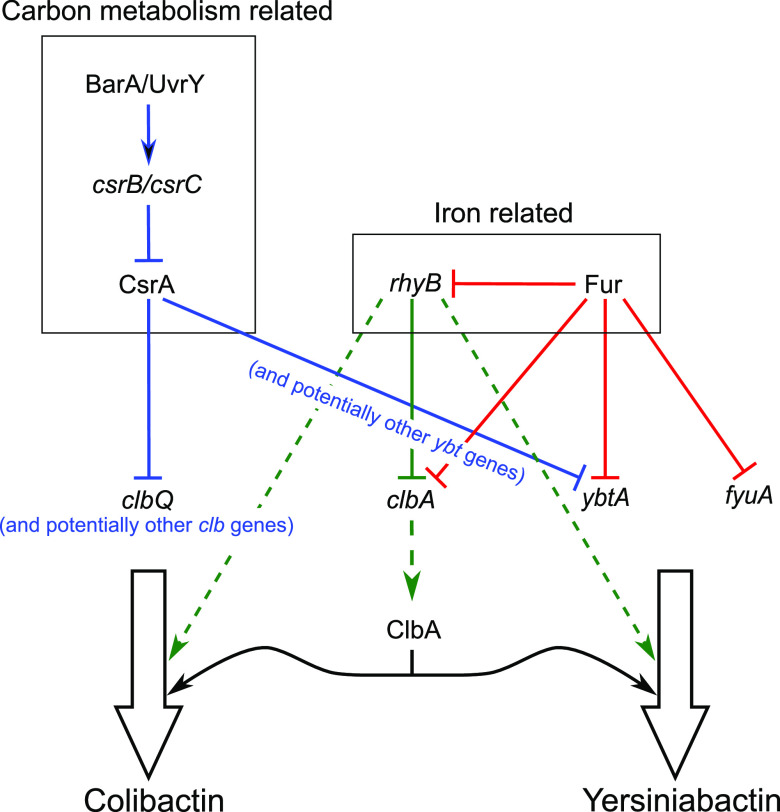
Integration of *pks* and HPI expression regulation into the Csr- and Fur-dependent regulatory networks. Colibactin and yersiniabactin expression both respond to central carbon- and iron-dependent regulation. CsrA inhibits at the posttranscriptional level expression of several genes of the colibactin and high pathogenicity island. The ferric uptake regulator (Fur) inhibits transcription of *clbA*, but also of *ybtA* and *fyuA*. The small regulatory noncoding RNA *ryhB* inhibits *clbA* expression at the posttranscriptional level. The *ryhB* regulatory RNA also modulates the efficiency of nonribosomal peptide biosynthesis via channeling of serine as a building block into colibactin and yersiniabactin biosynthesis (dashed arrow). Furthermore, the phosphopantetheinyl transferase ClbA activates polyketide synthases and nonribosomal peptide synthetases of both the colibactin as well as the yersiniabactin biosynthesis machinery.

In addition to iron availability, we show that the central metabolic state of the bacterial cells has a strong common impact on colibactin and yersiniabactin expression via direct dependence on the BarA/UvrY two-component system which is integrated into the carbon storage regulator signaling network (Fig. 7) and thus is closely linked to the central carbon metabolism. The sensor kinase BarA senses an appropriate environmental signal and transmits this signal by phosphorylating its cognate response regulator UvrY. Phosphorylated UvrY activates the transcription of the small noncoding RNA (sRNA) loci *csrB* and *csrC*. The sRNAs *csrB* and *csrC* compete with other RNA targets for CsrA binding, limiting the availability of this RNA-binding repressor (49, 55). CsrA is a global RNA-binding protein and a posttranscriptional regulator. It can either repress or enhance the expression of its RNA targets. CsrA has mostly been described as interacting with a specific sequence within the 5′ untranslated region (5′ UTR) of transcripts, with GGA being the minimal binding sequence (55, 56, 63). CsrA has also been reported to modulate BarA kinase activity as well as BarA-independent regulation of UvrY (49).

Fine-tuning gene expression is essential for the optimized production of complex metabolites, such as colibactin and yersiniabactin. Here, we demonstrate a direct link between primary and secondary metabolism by showing that the Csr system regulates colibactin and yersiniabactin production at the posttranscriptional level. We show that UvrY is required for the proper phenotypic expression of colibactin and yersiniabactin. Our study also provides evidence that functional CsrA is crucial to keep colibactin and yersiniabactin production in check via posttranscriptional repression of gene expression required for colibactin biosynthesis or regulation of colibactin expression. We have experimentally demonstrated that CsrA represses the expression of the thioesterase ClbQ. The additional presence of the CsrA binding motif in the upstream region of *clbL* and *clbS*, as well as in the coding sequence of four additional genes (*clbG*, *clbI*, *clbJ*, and *clbN*) involved in colibactin biosynthesis (Table 2), suggests that CsrA-dependent posttranscriptional regulation acts at multiple sites of the colibactin determinant. Similarly, we also detected multiple conserved CsrA binding sites in the yersiniabactin gene cluster upstream of *ybtA* and *fyuA* as well as within several coding regions (Table 3). Under iron limitation, the AraC-type regulator YbtA is required to initiate the expression of almost all HPI genes, which are subdivided into four transcriptional units (*ybtPQXS*, *ybtA*, *irp2-irp1-ybtUTE*, and *fyuA*), with the exception of its own gene (20, 64). The expression of *ybtA* is repressed by the global iron regulator Fur in an iron-rich environment (4, 60, 65, 66). Moreover, *ybtA* is subject to negative autoregulation (21), and two YbtA binding sites are located in the 5′ untranslated *ybtA* leader region. FyuA is the yersiniabactin receptor located in the outer membrane and is responsible for the import of iron-charged yersiniabactin. Its synthesis is strongly repressed by Fur and needs to be activated by YbtA (21, 64). Thus, CsrA-dependent posttranscriptional regulation adds another level of complexity to the already diverse checkpoints of colibactin and yersiniabactin expression. In this way, different signals, i.e., iron availability and central metabolic state, are integrated into the multilayered regulation of these fitness and pathogenicity factors.

The Csr system represents an important regulatory mechanism at the posttranscriptional level, which coordinates the expression of specific fitness and pathogenicity-associated traits with relevant physiological conditions. The RNA-binding regulatory protein CsrA is involved in the regulation of various cellular processes, including the synthesis of virulence factors in a wide range of bacteria (51, 67–71). CsrA-dependent regulation of iron uptake and siderophore expression has been reported in several pathogens, including Yersinia pseudotuberculosis, Legionella pneumophila, Salmonella enterica serovar Typhimurium, Pseudomonas aeruginosa, and also in Clostridium acetobutylicum (72–78). CsrA has been described to regulate the expression of the iron uptake system enterobactin in enteropathogenic E. coli (79). It has also been shown that siderophore synthesis and iron transport are regulated in Pseudomonas fluorescens and Vibrio fischeri by the UvrY homologue GacA (80, 81). The observation that the loss of UvrY resulted in a reduced expression of polyketide biosynthesis enzymes in Photorhabdus luminescens accompanied by decreased virulence in insects (82) further confirms that the Csr system plays an important role in controlling the expression of functionally different pathogenicity factors, including iron uptake systems and polyketides. Consequently, the Csr system is generally assumed to link carbon metabolism and iron uptake to optimize fitness during infection. This coupling is critical for the successful colonization or the establishment of an infection because it enables an adequate and fine-tuned modulation of bacterial gene expression in response to individual host environments and associated changes in nutritional demands, e.g., during the course of an infection.

Concerning colibactin and yersiniabactin, we describe a relationship between colocalization and coexpression. This “guilt-by-association” relationship (83) highlights that the encoded polyketides are coexpressed and are required for the same bacterial phenotype or trait. Both polyketides have been described as important fitness and pathogenicity factors in extraintestinal pathogenic E. coli (28, 29, 84, 85). Although different hypotheses regarding the biologically relevant function of colibactin exist, i.e., genotoxin/cyclomodulin versus bacteriocin (86), it is clear that the expression of colibactin and yersiniabactin can promote bacterial growth and survival *in vivo*, e.g., during infection. The production of these secondary metabolites and their corresponding large biosynthetic machineries also incurs high metabolic costs. As certain fitness factors provide important advantages in certain situations, the metabolic costs associated with them may be disadvantageous in microenvironments, in which they are not required. Accordingly, the precise regulation of gene expression in response to variable environmental signals is a prerequisite for bacterial adaptability, fitness, and pathogenicity in different habitats. This strategy is common in secondary metabolite production, as the complex and interconnected regulation of secondary metabolite expression by pathway-specific as well as global regulatory mechanisms has also been reported for various fungi (87). Against this background, our work emphasizes the importance of the interconnection between iron- and primary metabolism-responsive regulation of colibactin and yersiniabactin expression through the fine-tuned action of transcriptional regulators, such as Fur; posttranscriptional regulators, such as the *ryhB* sRNA; and the CsrA RNA-binding protein, as well as the posttranslational acyl group activation during colibactin and yersiniabactin biosynthesis via the PPTase ClbA.

## MATERIALS AND METHODS

### Bacterial strains, plasmids, genetic manipulations, and media.

Bacterial strains and plasmids used in this study are listed in Table 4 and 5, respectively.

**TABLE 4:** E. coli strains used in this study

Strain	Genotype and/or characteristics	Reference
E. coli		
536	pyelonephritis isolate 536; *pks*^+^, HPI^+^ (O6:K15:H31)	47
536-HDM	536Δ*hly*I, Δ*hly*II::*cat*	48
536-HDM *csrA*51	536-HDM *csrA*51::*cat*	This study
BL21 (DE3)	F-, *gal met r*-*m*-*hdsS* λ_lys_P*lacUV*5-T7-Gen1 P*lacI^q^lacI*	91
DH5α	F-*endA1 hsdR*17 *supE*44 *thi-1 recA1 gyrA*96 *relA*1 Δ(*argF*-*lacZYA*) *U169* (Φ60Δ*lacZ M*15*λ-*)	92
IHE3034	newborn-meningitis isolate; *pks*^+^, HPI^+^ (O18:K1:H7)	93
M1/5	Fecal isolate of a healthy individual; *pks*^+^, HPI^+^, Str^r^	42
M1/5 *csrA*51	M1/5 *csrA*153::*FRT-kan-FRT*; Kan^r^, Str^r^	This study
M1/5 Δ*uvrY*	M1/5 Δ*uvrY*::*FRT-cat-FRT*; Cm^r^, Str^r^	This study
M1/5 HPI-	M1/5 Δ(*ybtA-fyuA*)::*FRT*; Str^r^	This study
M1/5 HPI- *csrA*51	M1/5 Δ(*ybtA-fyuA*)::*FRT csrA*153::*FRT-kan-FRT*; Kan^r^, Str^r^	This study
M1/5 *Kan*-P*lacUV5*-*clbQ*'-'*venus*	M1/5 ΔP*clbQ*::(*FRT-kan-FRT*-P*lacUV5*) Δ*clbQ*4-723::(*venus-cat*); Cm^r^, Kan^r^, Str^r^	This study
M1/5 P*clbQ-*AL-*venus*	M1/5 Δ(*clbQ* 5′UTR-*clbQ*)::(artificial 5′UTR-*venus-cat*); Cm^r^, Str^r^	This study
M1/5 P*clbQ-*AL-*venus csrA*51	M1/5 Δ(*clbQ* 5′UTR-*clbQ*)::(artificial 5′UTR -*venus-cat*) *csrA*153:: *FRT-kan-FRT*; Cm^r^, Kan^r^, Str^r^	This study
M1/5 P*clbQ-clbQ*'-'*venus*	M1/5 Δ*clbQ*4-723::(*venus-cat*); *pks*^+^, HPI^+^, Cm^r^, Str^r^	This study
M1/5 P*clbQ-clbQ*'-'*venus csrA*51	M1/5 Δ*clbQ*4-723::(*venus-cat*) *csrA*153:: *FRT-kan-FRT*; Cm^r^, Kan^r^, Str^r^	This study
M1/5 P*lacUV*5-*clbQ*'-'*venus*	M1/5 ΔP*clbQ*::(FRT-P*lacUV*5) Δ*clbQ*4-723::(*venus-cat*); Cm^r^, Str^r^	This study
M1/5 P*lacUV*5-*clbQ*'-'*venus csrA*51	M1/5 ΔP*clbQ*::(FRT-P*lacUV*5) Δ*clbQ*4-723::(*venus-cat*) *csrA*153::*FRT-kan-FRT*; Cm^r^, Kan^r^, Str^r^	This study
M1/5 P*lacUV*5-clbQ*'-'*venus*	M1/5 ΔP*clbQ*::(*FRT*-P*lacUV*5) *ΔclbQ4-723*::(*venus-cat*); carries modified nucleotides in *clbQ* 5′UTR; Cm^r^, Str^r^	This study
M1/5 P*lacUV*5-clbQ*'-'*venus csrA*51	M1/5 ΔP*clbQ*::(*FRT-*P*lacUV*5) *ΔclbQ4-723*::(*venus-cat*) *csrA*153::*FRT-kan-FRT*; carries modified nucleotides in *clbQ* 5′UTR; Cm^r^, Kan^r^, Str^r^	This study
M1/5 P*lacUV*5-*ybtA*'-'*venus*	M1/5 ΔP*ybtA*::(FRT-P*lacUV*5) Δ*ybtA*4-960::(*venus-cat*); Cm^r^, Str^r^	This study
M1/5 P*ybtA-ybtA*'-'*venus*	M1/5 Δ*ybtA*4-960::(*venus-cat*); Cm^r^, Str^r^	This study
MG1655	K-12 F^–^ λ^–^ *ilvG*^–^ *rfb-50 rph-*1; *pks*^–^, HPI^–^	94
SP15	neonatal meningitis isolate, *pks*^+^, HPI^+^, Str^r^	95
SP15 Δ*uvrY*	SP15 Δ*uvrY*::*cat*; Cm^r^, Str^r^	This study
SP15 Δ*csrB*	SP15 Δ*csrB*::*cat*; Cm^r^, Str^r^	This study
SP15 Δ*csrC*	SP15 Δ*csrC*::*tet*; Str^r^, Tet^r^	This study
SP15 Δ*csrB* Δ*csrC*	SP15 Δ*csrB*::*FRT* Δ*csrC*::*tet*; Str^r^, Tet^r^	This study
SY327λ*pir*	λ(*lac pro*) *argE* (Am) *rif nalA recA*56 (λ*pir*)	96
Salmonella enterica Typhimurium		
WR1542	*fepA*::Tn*10*dTc, *iroN*::pGP704 *cir*::*MudJ* carrying plasmid pACYC5.3L; Ap^r^, Cm^r^, Kan^r^, Tc^r^	Gift from W. Rabsch, Wernigerode

**TABLE 5:** Plasmids used in this study

Plasmid	Features	Reference
pBAD33	Medium copy vector; p15A *araC* P*araBAD*; Cm^r^	97
pBAD33*	pBAD33; Δ*araC* ΔP*araBAD*	This study
pBAD33-*csrA*	pBAD33* with P*csrA*-*csrA*	This study
pBAD33-*venus*	pBAD33* with promoterless *venus*	45
pBAD33-*venus*-*csrA*	pBAD33* with P*csrA*-*csrA* and promoterless *venus*	This study
pCA9505	*gal uvrYC*; Ap^r^	50
pCA9505-*Mlu*I	pCA9505 with Δ*uvrY*	90
pCP20	temp-sensitive origin of replication, encodes Flp recombinase; Ap^r^, Cm^r^	98
pGEM-*csrC*	pGEM-T with *PcsrC-csrC*	This study
pKD3	Template plasmid for amplification of the FRT-flanked chloramphenicol resistance cassette; Ap^r^, Cm^r^	89
pKD4	Template plasmid for amplification of the FRT-flanked kanamycin resistance cassette; Ap^r^, Kan^r^	89
pKD46	Helper plasmid for l-arabinose inducible expression of λ-Red recombinase (*araC* P*araB*-*γ-β-exo*); Ap^r^	89
pKD46-*csrA*	pKD46 with P*csrA-csrA*	This study
pP*lacUV5*-AL-*venus*	pBAD33* with fusion of *lacUV5* promoter, artificial 5′UTR and *venus*	This study
pP*ybtA*AL*venus*	pBAD33* with fusion of *ybtA* promoter, artificial 5′UTR and *venus*	This study
pP*ybtA*-*ybtA*'-'*venus*2	pBAD33* with fusion of *ybtA* promoter, *ybtA* 5′UTR and *ybtA*(1-12)-*venus*	This study
pP*ybtA*-*ybtA*'-'*venus*2-*csrA*	pBAD33-*csrA* with fusion of *ybtA* promoter, *ybtA* 5′UTR and *ybtA*(1-12)-*venus*	This study
pP*ybtA*-ybtA*'-'*venus*2	pBAD33* with fusion of *ybtA* promoter, modified *ybtA* 5′UTR and *ybtA*(1-12)-*venus*	This study
pRS-*csrB*	pRS1553 with P*csrB-csrB*	This study
pUC-P*lacUV*5-*venus*	pUC18 template vector for amplification of fusion *lacUV5* promoter, artificial 5′UTR and *venus*; Ap^r^, Cm^r^	This study
pWKS30	Single copy vector; pSC101 origin of replication; *lacZα*; Ap^r^	99
pWKS-*csrA*	pWKS30 with P*csrA-csrA*	This study

Unless indicated otherwise, bacteria were grown in lysogeny broth (LB) (10 g · L^−1^ tryptone, 5 g · L^−1^ yeast extract, and 5 g · L^−1^ sodium chloride) or in a modified and glucose-free M9 medium (88) containing sodium pyruvate and casein hydrolysate (12 g · L^−1^ disodium hydrogen phosphate, 3 g · L^−1^ potassium dihydrogen phosphate, 3 g · L^−1^ casein hydrolysate, 2 g · L^−1^ sodium pyruvate, 1 g · L^−1^ ammonium chloride, 0.46 g · L^−1^ sodium chloride, 0.24 g · L^−1^ magnesium sulfate, 0.011 g · L^−1^ calcium chloride, and 0.2 mg · L^−1^ thiamine hydrochloride). For solid media, agar was used in concentrations of 16 g · L^−1^ in LB or 20 g · L^−1^ in M9. If required, 100 μM 2,2′-dipyridyl was added. The following antibiotics, if needed, were applied at the indicated concentrations: ampicillin, 100 μg · mL^−1^; chloramphenicol, 15 μg · mL^−1^ and 25 μg · mL^−1^ for low and medium copy number of the resistance cassette, respectively; kanamycin, 50 μg · mL^−1^; and tetracycline, 10 μg · mL^−1^.

Chromosomal genetic manipulations were carried out using the lambda red recombinase system according to Datsenko and Wanner (89). A detailed description of the construction of plasmids and mutants is found in the Supplemental Material. Unless otherwise indicated, E. coli strain DH5α was used as a host for the cloning of plasmids. Oligonucleotides used for strain manipulations and construction of plasmids are given in Table S1 in the supplemental material.

10.1128/mbio.03814-21.4 TABLE S1 Oligonucleotides used in this study. Download Table S1, PDF file, 0.2 MB. Copyright © 2022 Rehm et al. 2022 Rehm et al. https://creativecommons.org/licenses/by/4.0/ This content is distributed under the terms of the Creative Commons Attribution 4.0 International license.

### Megalocytosis and γH2AX assays.

HeLa cells, maintained by serial passage in Dulbecco’s modified Eagle’s medium (DMEM) supplemented with 10% fetal calf serum (FCS) and nonessential amino acids at 37°C and 5% CO_2_, were used to demonstrate the cytotoxic effect of colibactin on mammalian cells. Colibactin has been shown to induce double-strand breaks, which leads to cell cycle arrest and therefore the formation of megalocytotic cells (22). Assays to demonstrate megalocytosis and DNA damage induced by colibactin-producing bacteria were performed as described previously (22, 34). A detailed description is provided in the Supplemental Material.

### *N*-Myristoyl-d-asparagine (C14-asparagine) quantification.

*N*-Myristoyl-d-asparagine levels were quantified in cultures grown for 24 h in 9.5 mL DMEM-HEPES (Gibco). For details, see the Supplemental Material.

### DNA cross-linking assay.

The assay was performed as described previously (27). For details, please see the information provided in the Supplemental Material.

### Reporter gene measurements.

Fluorescence of the yellow fluorescent protein Venus was measured to determine the expression levels of various E. coli M1/5 *clbQ*- and *ybtA*-*venus* fusion strains. The reporter gene assay is described in detail in the Supplemental Material.

### RNA electrophoretic mobility shift assays (RNA EMSAs).

EMSAs with the purified CsrA protein and RNA molecules representing the 5′ untranslated leader regions of *clbQ* and *ybtA* were carried out using the LightShift chemiluminescent RNA EMSA kit (Thermo Fisher Scientific). For details, please see the Supplemental Material.

### Yersiniabactin quantification.

The amount of yersiniabactin produced by various E. coli strains was quantified using a reporter gene-based method that has been described in detail previously (42).

### Statistical analysis.

Statistical analyses were performed using the GraphPad Prism software (version 6.0). Figures show the mean values with standard deviation (STDEV.P). Either a one-way analysis of variance (ANOVA) followed by a Bonferroni posttest or Kruskal-Wallis followed by a Tukey test was applied unless stated otherwise. A *P* value of <0.05 was considered statistically significant, and *P* values are indicated by asterisks (*, *P* < 0.05; **, *P* < 0.01; ***, *P* < 0.001; ****, *P* < 0.0001).

10.1128/mbio.03814-21.5 TEXT S1 Detailed description of cloning procedures and experimental methods. Download Text S1, PDF file, 0.4 MB. Copyright © 2022 Rehm et al. 2022 Rehm et al. https://creativecommons.org/licenses/by/4.0/ This content is distributed under the terms of the Creative Commons Attribution 4.0 International license.
