# Supplementary material for: Two Polyketides Intertwined in Complex Regulation: Posttranscriptional CsrA-Mediated Control of Colibactin and Yersiniabactin Synthesis in Escherichia coli
Source: mBio. 2022 Feb 1;13(1):e03814-21. doi: 10.1128/mbio.03814-21 (PMC8805033; doi:10.1128/mbio.03814-21)
Supplement: FIG S1 [file mbio.03814-21-sf001.pdf]

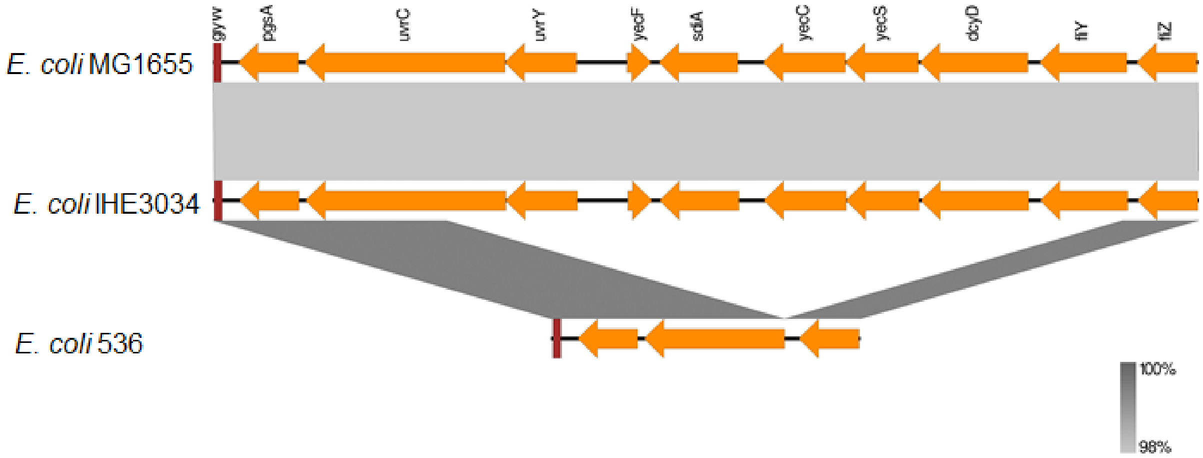

Figure S1. Comparison of the genomic region comprising *uvrY* in different *E. coli* strains. Analysis of the chromosomal region next to *uvrY* in the complete genome sequences of *pks* island-positive, but CPE-negative uropathogenic *E. coli* strain 536 with those of colibactin-producing newborn meningitis isolate IHE3034 as well as to K-12 laboratory strain MG1655 indicated that *E. coli* 536 carries a 6.1-kb chromosomal deletion spanning the region between *uvrC* and *fliY* and thus lacks the *uvrY* gene.
