# Supplementary material for: Two Polyketides Intertwined in Complex Regulation: Posttranscriptional CsrA-Mediated Control of Colibactin and Yersiniabactin Synthesis in Escherichia coli
Source: mBio. 2022 Feb 1;13(1):e03814-21. doi: 10.1128/mbio.03814-21 (PMC8805033; doi:10.1128/mbio.03814-21)
Supplement: FIG S2 [file mbio.03814-21-sf002.pdf]

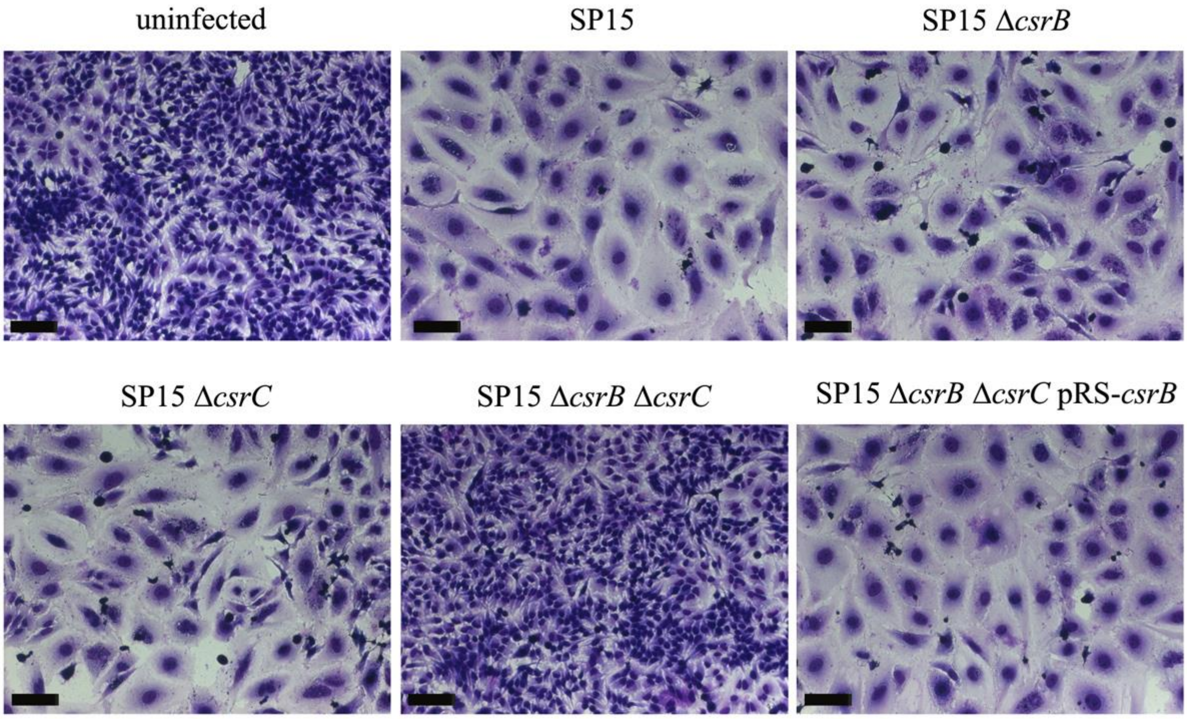

Figure S2. Impact of the small regulatory RNAs *csrB* and *csrC* on the cytopathic effect of *E. coli* strain SP15 in HeLa cells. HeLa cells were either not infected or infected with the indicated *E. coli* strains to a multiplicity of infection (MOI) of 250. After four hours of infection, HeLa cells were washed to remove bacteria and further cultivated. 72 h post infection, cells were washed and Giemsa-stained. Scale bars: 100 µm. Plasmid pRS-*csrB* was used to complement the *csrB* and *csrC* deletion in *E. coli* strain SP15Δ*csrB* Δ*csrC*.
