# Supplementary material for: Two Polyketides Intertwined in Complex Regulation: Posttranscriptional CsrA-Mediated Control of Colibactin and Yersiniabactin Synthesis in Escherichia coli
Source: mBio. 2022 Feb 1;13(1):e03814-21. doi: 10.1128/mbio.03814-21 (PMC8805033; doi:10.1128/mbio.03814-21)
Supplement: FIG S3 [file mbio.03814-21-sf003.pdf]

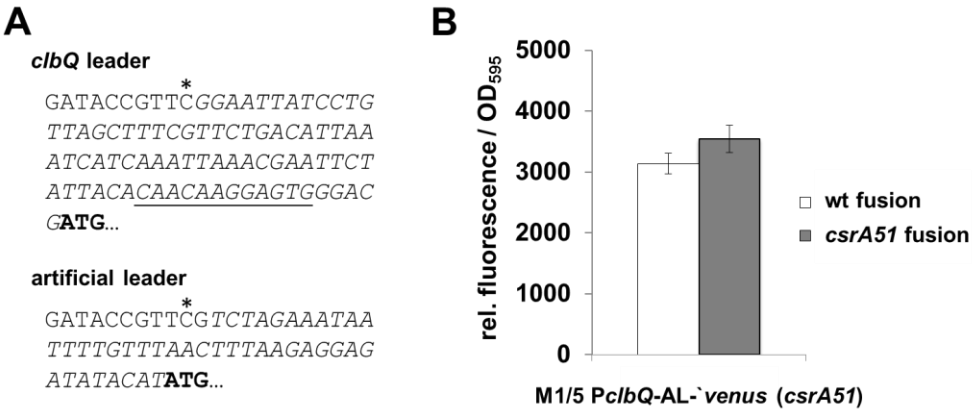

Figure S3. Activity of the *clbQ* promoter fused to a sequence encoding an artificial 5' unstranslated leader and *venus* as a reporter gene. A) Sequences encoding the *clbQ* 5' leader and the artificial leader are shown in italics, an asterisk represents the transcription start, the translational start codon is depicted in bold. The sequence encoding the putative CsrA binding motif is underlined. **B)** Expression from the *clbQ* promoter in *E. coli* strains M1/5 P*clbQ*-AL-'*venus* and M1/5 P*clbQ*-AL-'*venus* was measured by means of venus fluorescence after 10 h of growth in M9 minimal medium without glucose but containing pyruvate and casein hydrolysate. Mean values with standard deviations of three independent experiments are shown.
