# Supplementary material for: Two Polyketides Intertwined in Complex Regulation: Posttranscriptional CsrA-Mediated Control of Colibactin and Yersiniabactin Synthesis in Escherichia coli
Source: mBio. 2022 Feb 1;13(1):e03814-21. doi: 10.1128/mbio.03814-21 (PMC8805033; doi:10.1128/mbio.03814-21)
Supplement: TABLE S1 [file mbio.03814-21-st001.pdf]

Table S1. Oligonucleotides used in this study.

Designation	Sequence (5´ to 3´)	Purpose
**Plasmid clonings**		
csrA-XbaI-s	GCGC*TCTAGA*CAGCTGGGGTCGACAATTATC	cloning of pWKS-*csrA*
csrA-BamHI-as	GCGC*GGATCC*GGAAAGATTAGTAACTGGACTGCTG	cloning of pWKS-*csrA*
csrA-NcoI-s	GCGC*CCATGG*CAGCTGGGGTCGACAATTATC	cloning of pKD46-*csrA*
csrA-NcoI-as	GCG*CCCATGG*TTAGTAACTGGACTGCTGG	cloning of pKD46-*csrA*
pKD46-Seq1	ACGAGTATCGAGATGGCACA	control pKD46
pKD46-Seq2	ATGGCTCTTGTATCTATC	control pKD46
IHAPJPN126	GGGAATTCTCGTCTCCGTCAGTCAAAGC	cloning of pGEM-*csrC*
IHAPJPN127	GGGGATCCTGCGGCGGAATCTAACAG	cloning of pGEM-*csrC*
venus-fus-s	GGAGATATACATATGGTGTCTATCAC	*venus* template plasmid
venus-fus-as	GCGC*GTCGAC*TTACTTGTACAGCTCGTCCATG	*venus* template plasmid
P*lacUV5*-s	GCGC*GACGTC***CTCATTAGGCACCCCAGGCTTTACACTTTATGCTT CCGGCTCGTATAATGTGTGGA***TCTAGA*	*venus* template plasmid
P*lacUV5*-as	GATAGACACCATATGTATATCTCCTTCTTAAAGTTAAACAAAATTAT T*TCTAGA***TCCACACATTATACGAGC**	*venus* template plasmid
venus-XbaI	GCGC*TCTAGA*TGGTGTCTATCACTAAAGATCAAATC	*venus* cloning
MK67	CTTCTCTCATCCGCCAAAAC	*venus* cloning
venus-Seq-as	TACGTCGCCGTCCAGCTC	*venus* cloning
venus-Seq-as	TTCATGTGGTCGGGGTAGC	*venus* cloning
rrnB-XbaI-for	GCGC*TCTAGA*CAGAACGCAGAAGCGGTCTG	*rrnB* terminator cloning
rrnB-SacI-rev2	GCGC*GAGCTC*ACGCAAAAAGGCCATCCG	*rrnB* terminator cloning
ybtA-TLN-XbaI	GCGC*TCTAGA*CGGTGTAATCACCTGCCAG	*ybtA-venus* fusion
ybtA-long-for	GGAGATAACCAGGTCATGACGGAGTCAGTGTCTATCACTAAAG	*ybtA-venus* fusion

ybtA-long-rev	CTTTAGTGATAGACACTGACTCCGTCATGACCTGGTTATCTCC	*ybtA-venus* fusion
ybtA-int-up	CGCCAGTCCAGCCAGAAC	*ybtA-venus* fusion
ybtA-TLN-TTAT-for	CATTGGTCTAAGCCTTATGGAGATAAC	*ybtA-venus* fusion
ybtA-TLN-TTAT-rev	GACCTGGTTATCTCCATAAGGCTTAG	*ybtA-venus* fusion
ybtA-TXN-for	TGAGAATTAATGTGAATAATAACCATTATCTAGAAATAATTTTGTTTAAC	*ybtA-venus* fusion
**Genome modifications**		
ABU_*uvrY*::*cat*_Fw	acgaatgactaactatcagtagcgttatccctatttctggagatattccTGTAGGCTGGAGCTGCTTCG	*uvrY* deletion
ABU_*uvrY*::*cat*_Rv	tggctggctggttacggtttttaaaaacgcttttgcgtcaaactgatcacCCTCCTTAGTTCCTATTCCGAAG	*uvrY* deletion
csrA51-Del-M15-up	cgtaaatgccccgaaggaagtttctgttcaccgtgaagagatctaccagcgtatcTAAGTGTAGGCTGGAGCTGCTTC	*csrA* modification
csrA-Del-M15-rev	agagaaattttgagggtgcgtctcaccgataaagatgagacgcggaaagattaCATATGAATATCCTCCTTAGTTCC	*csrA* modification
ybtA-Del-for	cattcggttctatttatacccgcattggtctaagccacagggagataaccaggtcGTGTAGGCTGGAGCTGCTTC	partial deletion of HPI
fyuA-Del-rev	ctggttttaactagtggtgtaaaagggataccttttcggtatccccttttacaataaCATATGAATATCCTCCTTAGTTCC	partial deletion of HPI
clbQ-TLN1-*venus*-s	cattaaatcatcaaattaaacgaattctattacacaacaaggagtgggacgatgGTGTCTATCACTAAAGATCAAATCGTG	*clbQ-venus* fusion
clbQ-*cat*-as	cttttgatgatggaacagccatatctattgctccttgtatagttacacaactatttGCCTCAGGCATTTGAGAAGC	*clbQ-venus* fusion
Kan-P*lac*-fus-s	CCAGCCTACACCTCATTAGGCACCCCAGGC	*clbQ-venus* fusion
clbQ-P*lacUV5*-as	aattcgtttaatttgatgatttaatgtcagaacgaaagctaacaggataattccgTCCACACATTATACGAGCCG	*clbQ-venus* fusion
clbQ-P*lacUV5*-s	gagtaacagttggcgtggtaatagtgatgttccgcttgccgcagacaatcgtatcctcCATATGAATATCCTCCTTAGTTCC	*clbQ-venus* fusion

Kan-P*lac*-fus-as	CTAATGAGGTGTAGGCTGGAGCTGCTTCG	*clbQ-venus* fusion
clbQ-TLN-TTAT-*venus*-s	gctttcgttctgacattaaatcatcaaattaaacgaattctattacacattatggagtgggacgatgGTGTCTATCACTAAAGATCAAATC	*clbQ-venus* fusion
clbQ-TXN-*venus*-s	cttgactggcgttttatcttggtatggggtccatcgagcgtgttggcgataccgttcgTCTAGAAATAATTTTGTTTAACTTTAAG	*clbQ-venus* fusion
ybtA-TLN-*venus*-s2	ctcatctaccccattcggttctatttatacccgcattggtctaagccacagggagataaccaggtcatgGTGTCTATCACTAAAGATCAAATC	*ybtA-venus* fusion
ybtA-cat-as	gcccggatggggtatccgggcctctgtcagggaggagtttagggggggcgcgaccccggGCCTCAGGCATTTGAGAAGC	*ybtA-venus* fusion
ybtA-P*lacUV5*-s	gaggtgttgaacgcctgtaagcgcagaacaaaagttaagaccacgcagccgatcacCATATGAATATCCTCCTTAGTTCC	*ybtA-venus* fusion
ybtA-P*lacUV5*-as	ggcttagaccaatgcgggtataaatagaaccgaatggggtagatgagttattgaTCCACACATTATACGAGCCG	*ybtA-venus* fusion
**EMSA application**		
clbQ	CGGAAUUAUCCUGUUAGCUUUCGUUCUGACAUUAAAUCAUCAAAUUAAACGAAUUCUAUUACACAACAAGGAGUGGGACG	RNA-EMSA
ybtA	AUCAAUAACUCAUCUACCCCAUUCGGUUCUAUUUAUACCCGCAUUGGUCUAAGCCACAGGGAGAUAACCAGGUC	RNA-EMSA
phoB	GCAUUAAUGAUCGCAACCUAUUUAUUACAACAGGGCAAAUCAUG	RNA-EMSA

Restriction sites are written in italics. Homologous sequences to either the genome sequences for genome modifications or to a plasmid required for restriction-free cloning are indicated by lowercase letters. P*lacUV5* promoter sequences are highlighted in bold.
