# Supplementary material for: Two Polyketides Intertwined in Complex Regulation: Posttranscriptional CsrA-Mediated Control of Colibactin and Yersiniabactin Synthesis in Escherichia coli
Source: mBio. 2022 Feb 1;13(1):e03814-21. doi: 10.1128/mbio.03814-21 (PMC8805033; doi:10.1128/mbio.03814-21)
Supplement: TEXT S1 [file mbio.03814-21-s0001.pdf]

Detailed description of cloning procedures and experimental methods

The generation of plasmids and mutant strains is described below in detail. Strains and plasmids were verified by sequencing after cloning.

Plasmids

pWKS-*csrA*. The complementation vector pWKS-*csrA* was generated by amplification of *csrA* including app. 470 bp of its upstream region using *E. coli* IHE3034 as template and oligonucleotide pair *csrA*-XbaI-s/*csrA*-BamHI-as. The PCR product was digested with XbaI/BamHI and ligated with XbaI/BamHI digested pWKS30 to yield pWKS-*csrA*.

pKD46-*csrA*. The sequence of *csrA* and its promoter were amplified from *E. coli* IHE3034 with oligonucleotides *csrA*-NcoI-s and *csrA*-NcoI-as, digested with NcoI and ligated with NcoI digested pKD46.

Genome modifications

Chromosomal modifications in *E. coli* were carried out using the Lambda Red recombinase system of Datsenko & Wanner (1). Briefly, *E. coli* strains were first transformed with pKD46 (or pKD46-*csrA* for *csrA* modifications), grown in LB medium in the presence of 0.3 % L arabinose to induce expression of the Lambda Red recombinase encoded on pKD46 and then made competent for transformation with respective PCR products carrying the kanamycin or chloramphenicol cassette of pKD4 or pKD3, respectively. For this, oligonucleotides were designed in a way that PCR products carried nucleotide extensions of 50-60 bp homologous to the desired genomic site of manipulation. Clones were selected on agar containing either kanamycin (pKD4) or chloramphenicol (pKD3). After successful genomic manipulation of desired sequences, bacteria were cured from pKD46 (or pKD46-*csrA*) by serial streaking on LB agar and incubation at 37 °C. Successful curing was verified by PCR using oligonucleotide

pair pKD46-Seq1/pKD46-Seq2. If required, antibiotic resistance cassettes were removed by transformation with pCP20 (2), which encodes the FLP recombinase that cuts the resistance cassettes at the flanking FRT sites, thus leaving only the FRT site. Cells were cured from pCP20 by serial streaking on LB agar and incubation at 37 °C.

E. coli **strains M1/5Δ*uvrY*** and **SP15Δ*uvrY*** were obtained by transformation of pKD46 carrying *E. coli* M1/5 and SP15 cells with a PCR product comprising the *cat* cassette with extensions homologous to the upstream and downstream regions of *uvrY*. The PCR product was obtained by amplification of the chloramphenicol resistance cassette from pKD3 using primers ABU_*uvrY*::*cat*_Fw and ABU_*uvrY*::*cat*_Rv. Clones were selected on agar containing chloramphenicol.

E. coli **M1/5*csrA*51.** This strain was generated to synthesize a CsrA variant with reduced function by shortening of the protein by nine amino acids. It has been shown that the *E. coli* *csrA* gene is hard to manipulate (3). To facilitate chromosomal manipulation of *csrA* we modified the pKD46 plasmid, which is required for genomic modifications by the here-used methodology of Datsenko & Wanner (1), by introducing a *csrA* copy under the control of its native promoter, thus creating plasmid pKD46-*csrA*. Transformation of *E. coli* M1/5 with this plasmid did not only provide the cell with Lambda Red recombinase but also guaranteed expression of a functional CsrA protein during manipulation of the chromosomally encoded *csrA* copy. For manipulation of the genomic *csrA* copy, the kanamycin resistance cassette of pKD4 was amplified using oligonucleotide pair *csrA51*-Del-M15-up and *csrA*-Del-M15-rev. *E. coli* M1/5 pKD46-*csrA* was transformed with the PCR product, which was inserted in a way that a stop codon was placed after nucleotide 153 of the *csrA* sequence. Clones were selected on agar plates containing kanamycin.

E. coli **536-HDM *csrA*51** was generated from *E. coli* 536-HDM as described for *E. coli* M1/5*csrA*51.

***E. coli* M1/5 HPI⁻**, a partial HPI (high pathogenicity island coding for yersiniabactin) mutant (Δ*ybtA*-*fyuA*), was generated by transformation of M1/5 pKD46 with a PCR product obtained by amplification of the chloramphenicol resistance cassette of pKD3 using oligonucleotides *ybtA*-Del-for and *fyuA*-Del-rev. Clones were selected on agar plates containing chloramphenicol.

***E. coli* M1/5 HPI⁻ *csrA*51** was generated from *E. coli* M1/5 HPI⁻ as described for *E. coli* M1/5 *csrA*51.

***E. coli* strains SP15 Δ*csrB*, *E. coli* SP15 Δ*csrC*, *E. coli* SP15 Δ*csrB* Δ*csrC*.** lacking either the small regulatory RNA gene *csrB*, or *csrC* or both were generated by recombineering. The *csrB* gene was replaced by a *cat* cassette, whereas *csrC* was exchanged by a *tet* cassette. Clones were selected on agar plates containing chloramphenicol or tetracycline, respectively.

Construction of *venus* reporter strains and plasmids

pUC-P*lacUV*5-*venus*. This plasmid was generated in order to serve as a template for PCR reactions used in allelic replacements (1) to construct various chromosomally based *venus* reporter gene strains for *clbQ*, and *ybtA* (see below). The plasmid with pUC18 as a backbone contained the constitutive *lacUV*5 promoter, a sequence encoding an artificial 5'UTR leader derived from pTXB1 (lacking the LacI binding site), a modified *venus* sequence as a reporter gene and a chloramphenicol resistance cassette to enable selection of clones. The sequence of the artificial 5'UTR leader (from hereon designated artificial leader and abbreviated AL) was chosen because it neither did exhibit any binding site for transcriptional regulators nor did its transcript harbour a CsrA binding motif. Sequences of P*lacUV*5, AL and *venus* were fused by an overlap PCR. A first PCR product was obtained by amplification of the translational fusion of *rplL* and the *venus* gene from pMB*rplL*-*venus* using oligonucleotides *venus*-fus-s and

venus-fus-as introducing a downstream SalI restriction site. A second PCR product containing the P*lacUV*5, the artificial 5'UTR leader sequence and a portion of the *rplL-venus* (from hereon *venus*) sequence was yielded by template-free PCR using oligonucleotides P*lacUV*5-s and P*lacUV*5-as. This way, an AatII restriction site was introduced upstream of P*lacUV*5. The PCR products were then fused using oligonucleotides P*lacUV*5-s/*venus*-fus-as and the resulting amplicon was digested with AatII and SalI and ligated with AatII/SalI digested pMB*rplL-venus*, leading to plasmid pUC-P*lacUV*5-*venus.*

<u>*clbQ-venus* fusions</u>

A set of chromosomally based *clbQ-venus* fusions was constructed using allelic replacement described by Datsenko & Wanner (1). PCR products with extensions homologous to the *clbQ* locus were generated with pUC-P*lacUV*5-*venus* as a template.

E. coli* M1/5 P*clbQ-clbQ*'-'*venus. A translational fusion of the *clbQ* start codon and the *venus* sequence was obtained by amplification of the *venus* sequence and the chloramphenicol resistance cassette with oligonucleotides *clbQ*-TLN1-*venus*-s and *clbQ-cat*-as. *E. coli* M1/5 pKD46 was transformed with the resulting PCR product, *venus-cat*, to yield *E. coli* M1/5 P*clbQ-clbQ*'-'*venus*. This strain carried the native *clbQ* promoter as well as the native *clbQ* 5'UTR but the *venus* sequence instead of *clbQ*.

***E. coli* M1/5 P*clbQ-clbQ*'-'*venus csrA*51** was generated from *E. coli* M1/5 P*clbQ-clbQ*'-'*venus* as described above for *E. coli* M1/5*csrA*51.

E. coli* M1/5 P*lacUV*5-*clbQ*'-'*venus. To construct a *clbQ* leader fusion with the constitutive *lacUV*5 promoter, the native *clbQ* 5'UTR and *venus* as reporter, first the *clbQ* promoter of *E. coli* M1/5 was exchanged by the constitutive P*lacUV*5. Therefore, P*lacUV*5 from pUC-P*lacUV*5-*venus* was amplified with oligonucleotides *Kan*-P*lac*-fus-s and *clbQ*-P*lacUV*5-as. The resulting PCR product was fused downstream of the FRT site-flanked

kanamycin resistance cassette previously amplified from pKD4 using *clbQ*-P*lacUV*5-s and *Kan*-P*lac*-fus-as. The resulting PCR product, *Kan*-P*lacUV*5, carried nucleotide extensions homologous to the upstream region of the *clbQ* transcription start. After transformation of *E. coli* M1/5 (pKD46) this amplicon and subsequent selection on kanamycin containing agar plates the resulting strain, *E. coli* M1/5 Kan-P*lacUV*5-*clbQ*, was first transformed with pKD46 and afterwards with the *venus-cat* PCR sequence as described for *E. coli* M1/5 P*clbQ*-*clbQ*'-'*venus*, leading to *E. coli* M1/5 *Kan*-P*lacUV*5-*clbQ*'-'*venus*. Transformation with pCP20 led to the removal of the kanamycin resistance cassette and the final strain was designated to *E. coli* M1/5 P*lacUV*5-*clbQ*'-'*venus.*

***E. coli* M1/5 P*lacUV*5-*clbQ*'-'*venus csrA*51** was generated from *E. coli* M1/5 P*lacUV*5-*clbQ*'-'*venus* as described above for *E. coli* M1/5*csrA*51.

E. coli* M1/5 P*lacUV*5-*clbQ**'-'*venus was a derivative of *E. coli* M1/5 P*lacUV*5-*clbQ*'-'*venus* except that the sequence encoding the putative CsrA binding site within *clbQ* 5'UTR was changed from ACAAGGA to TTATGGA. With oligonucleotides *clbQ*-TLN-TTAT-*venus*-s and *clbQ-cat*-as the *venus-cat* sequence from pUC-P*lacUV*5-*venus* was amplified with extensions homologous to the region upstream and downstream of *clbQ*, respectively, introducing the modified sequence in the upstream extension. *E. coli* M1/5 *Kan*-P*lacUV*5-*clbQ* carrying pKD46 was transformed with this PCR amplicon and afterwards treated with pCP20 to remove the kanamycin resistance cassette, resulting in the desired strain *E. coli* M1/5 P*lacUV*5-*clbQ**'-'*venus.*

***E. coli* M1/5 P*lacUV*5-*clbQ**'-'*venus csrA*51** was generated from *E. coli* M1/5 P*lacUV*5-*clbQ**'-'*venus* as described above for *E. coli* M1/5*csrA*51.

***E. coli* M1/5 P*clbQ*-AL-'*venus*.** This strain carried a fusion of the *clbQ* promoter with the sequence encoding the artificial leader and the *venus* sequence of pUC-P*lacUV*5-venus being

amplified with oligonucleotides *clbQ*-TXN-*venus*-s and *clbQ-cat*-as. Transformation of *E. coli* M1/5 (pKD46) with this product yielded the desired strain.

***E. coli* M1/5 P*clbQ*-AL-'*venus csrA*51** was generated from *E. coli* M1/5 P*clbQ*-AL-'*venus* as described above for *E. coli* M1/5*csrA*51.

<u>*ybtA-venus* fusions</u>

Genome modifications. At first, a leader fusion of *ybtA* with the *venus* gene as a reporter was generated in the bacterial chromosomal using the method of Datsenko & Wanner (1). Chromosomal insertion of *venus* was performed in a way, that only the start codon of *ybtA* remained.

***E. coli* M1/5 M1/5 P*ybtA*-*ybtA*'-'*venus*.** In more detail, the *venus* encoding sequence attached to the chloramphenicol resistance cassette (*venus-cat*) of pUC-P*lacUV*5-*venus* was amplified with primer pairs *ybtA*-TLN-*venus*-s2/*ybtA-cat*-as. *E coli* M1/5 cells harbouring plasmid pKD46 for homologous recombination were transformed with the PCR product, plated on chloramphenicol containing agar and the resulting mutant strain M1/5 P*ybtA*-*ybtA*'-'*venus* was selected.

<u>Plasmids</u>

pBAD33*. A derivative of the medium copy vector pBAD33 devoid of the *araC* gene as well as the *araBAD* promoter, designated pBAD33*, was constructed to create plasmid-encoded *ybtA-venus* fusions. To generate this vector, pBAD33 was digested with SacI and EcoRV, treated with Mung Bean Nuclease (MBN) and religated. Cloning of the different *ybtA-venus* fusions into this vector is described below.

pBAD33-*csrA*. To obtain fusion plasmids that contained *csrA* for complementation, SacI/EcoRV digested pBAD33 was ligated with the *csrA* gene including app. 280 bp of its

upstream region previously cut from pWKS-*csrA*. The resulting plasmid was then digested with ZraI and EagI, treated with MBN and religated, yielding pBAD33-*csrA*.

pBAD33-*venus*-*csrA*. This plasmid, carrying a promoterless *venus* gene as well as the *crsA* gene with its own promoter, was generated by amplification of *venus* from pUC-P*lacUV5*-*venus* using primer pair *venus*-XbaI and MK67, subsequent digestion of the PCR product with XbaI and HindIII and ligation with XbaI/HindIII digested pBAD33-*csrA*.

pP*ybtA*-*ybtA*'-'*venus*2 and pP*ybtA*-*ybtA*'-'*venus*2-*csrA*. These plasmids represent fusions of the *ybtA* promoter, the *ybtA* 5'UTR and *venus* as a reporter. Since the *ybtA* promoter region does not only contain the *ybtA* promoter but also the divergently oriented *ybtP* promoter, a transcription terminator had to be introduced upstream of the *ybtA* promoter to prevent expression from the *ybtP* promoter. Therefore, the *rrnB* terminator was cloned into pBAD33* and pBAD33-*csrA*. The terminator was amplified from pBAD33 using primer pair *rrnB*-XbaI-for/*rrnB*-SacI-rev2, digested with XbaI and SacI and ligated with XbaI/SacI digested pBAD33* or pBAD33-*csrA* leading to pBAD33-*rrnB* or pBAD33-*csrA*-*rrnB*, respectively. A sequence harbouring the *ybtA* promoter region, the *ybtA* 5'UTR as well as the *venus* gene, was amplified from *E. coli* M1/5 P*ybtA*'-'*ybtA*-*venus* using primer pair *ybtA*-TLN-XbaI and MK67, digested with XbaI and SphI and ligated with XbaI/SphI pBAD33-*rrnB*. The resulting plasmid pP*ybtA*-*ybtA*'-'*venus*1 contained a translational fusion of the first three nucleotides of *ybtA* and the *venus* gene. In an additional step, the *ybtA* sequence was extended from three to twelve nucleotides in a restriction-free cloning strategy (Unger et al., 2010) using pP*ybtA*-*ybtA*'-'*venus*1 as a template and *ybtA*-long-for and *ybtA*-long-rev as primers, yielding plasmid pP*ybtA*-*ybtA*'-'*venus*2. Plasmid pP*ybtA*-*ybtA*'-'*venus*2-*csrA* was generated by digestion of pP*ybtA*-*ybtA*'-'*venus2* with XbaI and SphI and cloning of the P*ybtA*-*ybtA*'-'*venus* sequence into XbaI/SphI digested pBAD33-*csrA*-*rrnB*.

pP_ybtA-ybtA_*'-'_venus_2. The sequence encoding the putative CsrA binding motif within the *ybtA* leader of pP*ybtA*-*ybtA*'-'*venus*2 was modified on DNA basis from ACAGGGA to TTATGGA using the respective plasmids as templates with the primer pair *ybtA*-TLN-TTAT-for/*ybtA*-TLN-TTAT-rev by restriction-free cloning. The resulting plasmid was designated pP_ybtA_-_ybtA_*'-'_venus_2.

pP*lacUV*5-AL-*venus*. A plasmid carrying a fusion of the *lacUV5* promoter, a sequence encoding the 5' untranslated region without CsrA binding motif (artificial leader, AL) and the *venus* sequence and was designed to be used as a control for CsrA-independent regulation of various *ybtA*- and *fyuA*-*venus* fusions. In a first step, the P*lacUV*-AL fusion with a partial venus sequence was amplified from template vector pUC-P*lacUV*5-*venus* with primers *Kan*-P*lac*-fus-s and *venus*-Seq-as. In a second step, this PCR product served as a megaprimer and plasmid pP*lacUV*5-*ybtA*'-'*venus*1 as a template in a restriction-free cloning approach as described above to generate pP*lacUV*5-AL-*venus*.

pP*ybtA*-AL-*venus*. This plasmid carried a fusion of the *ybtA* promoter, a sequence encoding the 5' untranslated region without CsrA binding motif (artificial leader, AL) and the *venus* sequence. A megaprimer pair was generated via PCR using pP*lacUV*5-AL-*venus* as a template and primers *ybtA*-TXN-for and *venus*-Seq-as2. Next, these megaprimers were used to replace the *ybtA* 5'UTR of plasmid p*PybtA*-*ybtA*'-'*venus*2 by the AL using restriction-free cloning as described above, leading to pP*ybtA*-AL-*venus*.

Megalocytosis and γH2AX assays

HeLa cells, maintained by serial passage in DMEM supplemented with 10 % FCS and non-essential amino acids at 37 °C and 5 % CO_2, were used to demonstrate the cytotoxic effect of colibactin on mammalian cells. Colibactin has been shown to induce double strand breaks, which leads to cell cycle arrest and therefore formation of megalocytotic cells (4).

For assays to demonstrate megalocytosis induced by colibactin-producing bacteria, 2.5 x 10^4 HeLa cells per well were seeded in a 24-well cell culture plate. To examine the extent of DNA double strand breaks using serine139-phosphorylated H2AX, γH2AX, induced by bacteria synthesizing colibactin, 1.25 x 10^5 HeLa cells were dispersed in a 6-well cell culture plate. The next day, bacterial overnight cultures were used to infect HeLa cells with a multiplicity of infection (MOI) of 200 in interaction medium (DMEM, 5 % FCS, 25 mM HEPES). Four hours post infection, cells were washed 4-8 times with 1x Hank´s Balanced Salt Solution (HBSS) (Thermo Fisher Scientific, Wesel, Germany) containing 100 µg mL^{-1} gentamycin and further incubated at 37 °C and 5 % CO_2 in DMEM with 10 % FCS, non-essential amino acids and 200 µg mL^{-1} gentamycin (600 µg mL^{-1} gentamycin for cells infected with *E. coli* strains carrying the *csrA*51 mutation).

For demonstration of megalocytosis, cells were washed twice with 1x HBSS 72 h post infection, afterwards fixed with methanol, stained with 10 % Giemsa stain (Sigma-Aldrich, Munich, Germany) and then used for microscopy.

For γH2AX detection, cells were washed eight hours post infection with ice-cold phosphate buffered saline (137 mM sodium chloride, 2.7 mM potassium chloride, 10.1 mM disodium hydrogen phosphate, 1.8 mM potassium dihydrogen phosphate, pH 7.4) and immediately suspended and lysed with sample buffer (32.9 mM Tris, 15 % (v/v) glycerol, 0.5 % sodium dodecyl sulfate, pH 6.8). Total protein in cell extracts was quantified using BCA reagent (Thermo Fisher Scientific). 4-5 µg total protein were first separated by SDS-PAGE using 4-20 % Mini-Protean-TGX gels (Bio-Rad, Munich, Germany) and then transferred onto a

polyvinyl difluoride (PVDF) membrane. For the detection of γH2AX antibody anti-γH2A.X (phospho S139) antibody (Abcam, Cambridge, UK) and as secondary antibody Peroxidase AffiniPure goat anti-rabbit IgG (H+L) (Dianova, Hamburg, Germany) were used. Alternatively, anti-phospho-Histone H2A.X (Ser139), clone EP854(2)Y (Merck-Millipore, Darmstadt, Germany) served as a primary antibody for detection of γH2AX. As a control, β-actin was detected with anti-beta actin [AC-15] antibody (HRP) (Abcam). As substrate for horseradish peroxidase Bio-Rad Clarity Western ECL-substrate was used (Bio-Rad). Blots were analysed using the ChemiDoc XRS+ system (Bio-Rad).

N-myristoyl-D-asparagine (C14-Asparagine) quantification

Cultures in 9.5 mL DMEM-Hepes (Gibco) inoculated with 500 µL o/n LB culture were used for lipid extraction. After 24 h, the OD_{600} was measured and the culture was centrifuged for 5 min at 5000 × g and 4 °C. Pellets were resuspended in 500 µL HBSS and frozen at -80 °C until lipid extraction.

Pellets were crushed in Lysing Matrix A tubes (MP biomedicals, Santa Ana, California, USA) with a Precellys instrument (5 m/s; 2 x 30 s) (Ozyme, Montigny le Bretonneux, France) after addition of 5 µL internal standard (IS) mixture (Deuterium-labeled compounds: 400 ng/mL). After two crush cycles (5 m/s, 30 s), 0.3 mL of cold methanol (MeOH) was added and samples were centrifuged at 1016 × g for 15 min (4 °C). HLB plates (OASIS® HLB 2 mg, 96-well plate, Waters, Ireland) were used for solid phase extraction of lipids after being conditioned with 0.5 mL MeOH and 0.5 mL H_2O/ MeOH (90:10, v/v). Samples' volumes were brought to 2 mL with water and then loaded on the plate at a flow rate of about one drop per 2 s. After complete loading, columns were washed with 0.5 mL H_2O/ MeOH (90:10, v/v) and dried under aspiration. Lipids were eluted with 750× µL MeOH and extracts were submitted to two series of evaporation under N_2, then resuspended in a final volume of 10 µL MeOH for quantification of C14-Asn.

The quantification of C14-Asn was performed by the MetaToulLipidomics Facility (INSERM UMR1048, Toulouse, France). They developed a quantification assay by high-performance liquid chromatography tandem mass spectrometry analysis (LC-MS/MS) (5). Briefly a high-performance liquid chromatography (HPLC, Agilent 1290 Infinity) was designed to separate the metabolites using a C18 column. The HPLC system was coupled online to an Agilent 6460 triple quadrupole MS for detection and quantification (Agilent Technologies). Analyses were performed in Multiple Reaction Monitoring in optimal conditions. Peak detection, integration and quantitative analysis were done using Mass Hunter Quantitative analysis software (Agilent Technologies).

DNA crosslinking assay

The assay was performed as previously described (6). Linearized plasmid DNA was produced by digestion of pUC19 plasmid with BamHI (New England Biolabs). For bacteria-DNA interactions, 6 x 10^6 CFU were cultivated in 500 µL micro tube under static condition at 37 °C. After 3.5 h of cultivation, 400 ng of linearized DNA and EDTA of a final concentration of 1mM were added and further incubated for 30 min. Following centrifugation for 5 min at 5,000 × g to pellet bacteria, the DNA present in the supernatants was purified using the PCR Purification Kit (Qiagen) according to the manufacturer’s recommendations.

A denaturing 1% agarose gel was prepared in a 100 mM NaCl and 2 mM EDTA solution (pH 8.0). The gel was then soaked overnight in an alkaline running buffer solution (40 mM NaOH and 1 mM EDTA, pH ~12.0). 100 ng of each DNA sample were loaded on the denaturing agarose gel. The gel was run for 45 min at 1 V/cm and then for 2 h at 2 V/cm and then neutralized for a total of 45 min in a 100 mM Tris pH 7.4 buffer solution containing 150 mM NaCl, changed frequently. The gel was stained with GelRed and revealed with UV exposure using the ChemiDoc Imaging System (BioRad).

Reporter gene measurements

Fluorescence of the yellow fluorescent protein Venus was measured to determine the expression levels of various *E. coli* M1/5 *clbQ*- and *ybtA*-*venus* fusion strains.

For *clbQ*-*venus* reporter gene assays, 50 mL glucose-free M9 medium with pyruvate and casein hydrolysate were inoculated to an OD_{600} of 0.05 from overnight cultures of respective strains as well as their controls (corresponding strains without the cloned *venus* gene). For strains carrying plasmids 100 µg mL^{-1} ampicillin was added. During growth at 37 °C under shaking conditions, samples were drawn to determine the OD_{600} for monitoring of growth and to measure fluorescence. Samples were centrifuged and washed once with phosphate buffered saline (PBS; 137 mM sodium chloride, 2.7 mM potassium chloride, 10.1 mM disodium hydrogen phosphate, 1.8 mM potassium dihydrogen phosphate, pH 7.4) and resuspended in PBS. 150 µl of each sample were transferred into a black 96-well polystyrene microplate with transparent and flat bottom (Greiner Bio-One, Bad Nenndorf, Germany) and fluorescence was determined in a Tecan Infinite 200 Reader (Tecan Group Ltd., Männedorf, Switzerland) with following parameters: 10 s shaking with an amplitude of two, absorption was measured at 595 nm (10 nm bandwidth) and five flashes (pulses), fluorescence was excited at 485 nm (20 nm bandwidth) and emitted at 535 nm (25 nm bandwidth) with 50x amplification, 10 flashes and 20 µs of integration time.

E. coli M1/5 strains carrying plasmids with *ybtA*-*venus* fusions were grown in glucose-free M9 medium with pyruvate and casein hydrolysate in the presence of 100 µM 2,2'-dipyridyl to induce iron limitation, and 15 µg mL^{-1} chloramphenicol. For each strain (including a strain with a promoterless *venus* gene as negative control) two wells of 150 µl fresh medium were inoculated to an OD_{600} of 0.05 from overnight cultures. Cells were grown for 23 h in a Tecan Infinite 200 Reader (Tecan Group Ltd.) with a shaking amplitude of 2 and the parameters as

described for *clbQ-venus* fusion strains, except that absorbance and fluorescence were determined in 15 min-intervals with a 3-min shaking pause during each interval. For the measurement of fusion strains containing the *ybtA* or *lacUV5* promoter fused to the sequence encoding an artificial leader and the *venus* gene a 40x amplification of the fluorescence signal was applied.

Raw data were first corrected for autofluorescence of PBS (*clbQ-venus*) or M9 medium (*ybtA-venus*), respectively and the resulting values were then normalized to the OD_{595}. The calculated fluorescence/OD_{595} values of the negative control strains were subtracted from that of the respective fluorescent strains to correct for autofluorescence of bacteria.

<u>RNA electrophoretic mobility shift assays (RNA EMSA)</u>

Biotin-labelled and unlabeled RNA oligonucleotides representing the 5' untranslated leader transcripts of *clbQ*, *ybtA* and *phoB* as a negative control (7) were purchased from metabion GmbH (Planegg/Steinkirchen, Germany). Purified recombinant CsrA protein of *E. coli* (catalog # MBS1176282) was purchased from MyBioSource. EMSA were carried out using the LightShift™ Chemiluminescent RNA EMSA Kit (Thermo Fisher Scientific). Briefly, 10 µl reaction mixtures containing biotin-labeled RNA and increasing amounts of purified CsrA in incubation buffer (10 mM HEPES, 20 mM potassium chloride, 1 mM magnesium chloride, 1 mM DTT, 10 µg yeast tRNA (Thermo Fisher Scientific), 40 units RNaseOUT™ (Thermo Fisher Scientific), pH 7.3) were prepared and incubated for 30 min at 37 °C to enable protein binding to RNA. For competitive EMSA, non-labeled RNA was added to the labelled RNA. After incubation, the samples were immediately loaded on precast native 8 % Tris Borate EDTA polyacrylamide gels (anamed Elektrophorese GmbH, Groß-Bieberau/Rodau, Germany) and run for 90 min 0.5x TBE and 80 V. Afterwards, samples were transferred onto a positively charged nylon membrane in 0.5x TBE and 300 mA for 37 min, which was followed by RNA crosslinking to the membrane using UV light (2x with 1,200 units). Biotinylated RNA was

detected with the chemiluminescent detection module (Thermo Fisher Scientific) according to the manufacturer´s protocol and blots were analyzed in a ChemiDoc XRS+ System (Bio-Rad, Munich, Germany).

Yersiniabactin quantification

The amount of yersiniabactin produced by various *E. coli* strains was quantified using the *Salmonella* indicator strain WR1542 carrying the plasmid pACYC5.3L (gift from W. Rabsch, Wernigerode). This plasmid carries all necessary genes of the high pathogenicity island for uptake of yersiniabactin, *fyuA*, *ybtA*, *irp6*, *irp7* and *irp8*. Moreover, the plasmid harbors a transcriptional fusion of the *fyuA* promoter with the luciferase gene as a reporter allowing the detection of yersiniabactin-dependent upregulation of the *fyuA* promoter, thus indirect quantification of yersiniabactin. The method has been previously described in detail (8).

References

1. Datsenko KA, Wanner BL. 2000. One-step inactivation of chromosomal genes in *Escherichia coli* K-12 using PCR products. Proc Natl Acad Sci USA 97:6640-5.
2. Cherepanov PP, Wackernagel W. 1995. Gene disruption in *Escherichia coli*: TcR and KmR cassettes with the option of Flp-catalyzed excision of the antibiotic-resistance determinant. Gene 158:9-14.
3. Timmermans J, Van Melderen L. 2009. Conditional essentiality of the *csrA* gene in *Escherichia coli*. J Bacteriol 191:1722-4.
4. Nougayrède JP, Homburg S, Taieb F, Boury M, Brzuszkiewicz E, Gottschalk G, Buchrieser C, Hacker J, Dobrindt U, Oswald E. 2006. *Escherichia coli* induces DNA double-strand breaks in eukaryotic cells. Science 313:848-51.
5. Pérez-Berezo T, Pujo J, Martin P, Le Faouder P, Galano JM, Guy A, Knauf C, Tabet JC, Tronnet S, Barreau F, Heuillet M, Dietrich G, Bertrand-Michel J, Durand T, Oswald E, Cenac N. 2017. Identification of an analgesic lipopeptide produced by the probiotic *Escherichia coli* strain Nissle 1917. Nature Commun 8:1314.
6. Bossuet-Greif N, Vignard J, Taieb F, Mirey G, Dubois D, Petit C, Oswald E, Nougayrède JP. 2018. The colibactin genotoxin generates DNA interstrand cross-links in infected cells. mBio 9.
7. Jonas K, Edwards AN, Ahmad I, Romeo T, Romling U, Melefors O. 2010. Complex regulatory network encompassing the Csr, c-di-GMP and motility systems of *Salmonella* Typhimurium. Environ Microbiol 12:524-40.
8. Martin P, Marcq I, Magistro G, Penary M, Garcie C, Payros D, Boury M, Olier M, Nougayrède JP, Audebert M, Chalut C, Schubert S, Oswald E. 2013. Interplay between siderophores and colibactin genotoxin biosynthetic pathways in *Escherichia coli*. PLoS Pathog 9:e1003437.
